# Transcriptomic signatures in whole blood of patients who acquire a chronic inflammatory response syndrome (CIRS) following an exposure to the marine toxin ciguatoxin

**DOI:** 10.1186/s12920-015-0089-x

**Published:** 2015-04-02

**Authors:** James C Ryan, Qingzhong Wu, Ritchie C Shoemaker

**Affiliations:** ProteoGenomics, LLC, Vero Beach, FL 32963 Florida; NOAA Center of Excellence for Oceans and Human Health at Hollings Marine Laboratory, Charleston, SC USA; Center for Research on Biotoxin-Associated Illnesses, Pocomoke, MD USA

**Keywords:** Ciguatera, Gene expression, HLA, Ciguatoxin, Immune, Chronic inflammation, Chronic illness, Transcriptomics

## Abstract

**Background:**

Ciguatoxins (CTXs) are polyether marine neurotoxins found in multiple reef-fish species and are potent activators of voltage-gated sodium channels. It is estimated that up to 500,000 people annually experience acute ciguatera poisoning from consuming toxic fish and a small percentage of these victims will develop a chronic, multisymptom, multisystem illness, which can last years, termed a Chronic Inflammatory Response Syndrome (CIRS). Symptoms of ciguatera CIRS include fatigue, cognitive deficits, neurologic deficits, pain and sensitivity to light. There are few treatment options for ciguatera CIRS since little is known about its pathophysiology.

**Methods:**

This study characterizes the transcriptional profile in whole blood of 11 patients with ciguatera-induced CIRS and 11 normal controls run in duplicate using Agilent one color whole genome microarrays. Differential expression was determined by using a combination of moderated *t*-test p-value and fold change (FC). Significant genes were subjected to gene ontology, principal component analysis and SVM classification. Seven significant genes found by microarray were validated by PCR.

**Results:**

Using a low stringency (p < 0.05 and FC > 1.4) and a high stringency (p < 0.01 and FC > 1.5) filter, the resulting gene sets of 185 and 55, respectively, showed clear separation of cases and controls by PCA as well as 100% classification accuracy by SVM, indicating that the gene profiles can separate patients from controls. PCR results of 7 genes showed a 95% correlation to microarray data. Several genes identified by microarray are important in wound healing (CD9, CD36, vWF and Factor XIII), adaptive immunity (HLA-DQB1, DQB2, IL18R1 and IL5RA) and innate immunity (GZMK, TOLLIP, SIGIRR and VIPR2), overlapping several areas shown to be disrupted in a mouse model of acute exposure to ciguatoxin. Another area of interest was differential expression of long, non-coding sequences, or lncRNA.

**Conclusions:**

Disruptions of innate and adaptive immune mechanisms were recorded at both the genomic and proteomic level. A disruption in the HLA-T cell receptor axis could indicate HLA haplotype sensitivity for this chronic syndrome, as noted in many autoimmune conditions. Taken together, these indicators of illness provide additional insights into pathophysiology and potential therapies.

**Electronic supplementary material:**

The online version of this article (doi:10.1186/s12920-015-0089-x) contains supplementary material, which is available to authorized users.

## Background

Ciguatera fish poisoning (CFP) is the most common marine toxin poisoning worldwide, with estimates of 50,000-500,000 cases annually [1]. Precise numbers of cases are nearly impossible to obtain due to widespread underreporting and the fact that no clinical diagnostic test exists to verify the acute illness, which may result from fish contaminated with as little as 0.1 ppb ciguatoxin [2]. Produced in tropical and subtropical oceans by benthic dinoflagellates of the genus *Gambierdiscus*, ciguatoxins (CTXs) are found in numerous reef fish species, such as barracuda, grouper and snapper, with larger and older fish usually being the most toxic. As reviewed by Lewis [3], families of ciguatoxins have been isolated from the Pacific and Indian Oceans, along with the Caribbean Sea, and suites of CTX congeners have been distinguished by minor differences in their cyclic polyether backbone and different toxicities. These heat stable, lipid soluble, cyclic polyether toxins are potent activators of voltage-gated sodium channels, and the consumption of toxic levels is characterized by acute gastrointestinal and neurological symptoms, including vomiting, diarrhea, abdominal pain, headache, tachycardia, prostration, severe localized itching, tingling of extremities and lips, and temperature reversal [4]. While most of these symptoms are self-healing in a matter of weeks, other symptoms of CFP can last several years [2]. These long term symptoms can include fatigue, pain, weakness, depression and cognitive deficits, as well as hypersensitivity to inflammagen exposure [5].

Ciguatoxin is slowly metabolized in mammals after ingestion. In rats, the plasma terminal half life was estimated to be 82 and 112 hours after oral and intraperitoneal administration, respectively, with the main route of excretion through the feces [6]. While there are few treatment options for CFP, most symptoms subside after a few weeks, similar to the expected clearance of toxin. Herbal remedies and the osmolyte mannitol have shown limited efficacy if administered in an appropriate time window [7,8]. It is still unknown why a small percentage of acute poisonings, roughly 5%, transition into a chronic illness characterized by a persistent inflammatory syndrome. This syndrome, Chronic Inflammatory Response Syndrome (CIRS), can be identified by serum protein abnormalities in transforming growth factor beta-1 (TGFβ-1), split product of complement component 4 (C4a), vasoactive intestinal peptide (VIP) and others [5].

Transcriptomic studies of acute CTX exposure were conducted in mice with the most potent congener, P-CTX-1, and followed the toxicogenomic effects over a 24 hour period [9-11]. These studies examined three different organ systems in parallel, the immune system using whole blood, the central nervous system using the brain, and detoxification mechanisms using the liver. Although the mouse model system only studied the acute response, and was confounded by a pronounced hypothermic response to the toxin, some of the molecular pathways that were disrupted were similar to those in patients with ciguatera-induced CIRS, or CIRS-ciguatera, including genes for complement and hemostasis. In mice, over half of the 15 differentially expressed genes shared by all three tissues had functions directly involving the immune system, such as cytokines, interleukins and immunoglobulin related genes [10]. Additionally, expression of inflammatory cytokines was shown in a murine macrophage cell line upon direct exposure to P-CTX-1B [12].

There is neither a treatment nor a test for acute ciguatoxin exposure, yet the effects are often debilitating and on rare occasions, even deadly. Use of a CIRS treatment protocol shows promise in chronic cases [5]. Although proteomic abnormalities are documented in patients with CIRS-ciguatera, it is relatively difficult to measure protein expression on a global scale and the future discovery of proteomic abnormalities may be slow. Meanwhile, advances in genomics technology make surveying the expression of the genome fairly routine. We have evidence that the immune system plays a role in responding to this toxic exposure, as seen in both humans and mice [5,11]. To gain an understanding of CIRS-ciguatera, this study uses a top down approach, looking globally at immune perturbations through gene expression and finding elements implicated in pathophysiology. By focusing on global gene expression in the blood, we hope to broaden our understanding of dysfunctional systems, and encourage a focus on the proteomic discovery of biomarkers and guideposts for treatment protocols in these patients.

## Methods

### Subjects

Participation in this study was voluntary and informed consent was obtained from case and control subjects to use their data for research purposes. Institutional Review Board (IRB) approval for this study was granted by Copernicus Group IRB, LLC, Research Triangle Park, NC. Medical history and symptoms registries were recorded for cases and controls, as described in detail previously [5]. Patient samples came from two separate medical clinics and were considered as potential CIRS-ciguatera cases (N = 11) if they were considered to have (1) developed an acute illness typical of ciguatera within 24 hours of reef fish consumption (typical symptoms can be found in [4,13]), (2) no confounding illnesses and (3) symptoms that persisted beyond six months. Acquisition of illness data as well as patient symptoms over the course of illness can be found in Additional file 1: Tables S1 and Additional file 2: Table S2. Age matched volunteers (N = 11) served as normal controls if they had no (1) current illness of any kind requiring medical treatment, (2) history of acute multisystem illness following fish consumption, or multisystem, multisymptom illness following exposure to environmentally produced biotoxins or (3) untreated chronic illness. Patients meeting initial inclusion criteria received a physical examination, proteomic and transcriptomic blood analyses.

### Serum protein labs

All patients reported in this work met the same case definition for CIRS-ciguatera as described previously in a larger proteomic study [5]. Nine of the eleven patients in the current study were part of the former study, with two new patients added here. Proteomic methods are presented only to disclose parameters for positive diagnosis. For results of proteomics, please refer to [5]. The diagnosis of CIRS-ciguatera was made using the standard process of differential diagnosis, including the assessment of exposure risks, symptoms and blood lab results that included abnormalities in serum protein markers. Briefly, patients must have presented with abnormal results in at least four of the eight objective serum markers found most informative for CIRS, which are TGFβ1, VIP, MSH, MMP9, C4a, VEGF, ACTH/cortisol and ADH/osmolality.

Laboratory blood tests were performed by CLIA-licensed facilities, LabCorp, Quest Diagnostics, National Jewish Center (Denver) and Cambridge Biomedical. Testing included alpha melanocyte stimulating hormone (MSH), VIP, matrix metalloproteinase 9 (MMP9), split products of complement component 3 (C3a) and C4a, TGFβ-1, vascular endothelial growth factor (VEGF), lipid profile, complete blood count (CBC), comprehensive metabolic panel (CMP), gamma-glutamyl transpeptidase (GGTP), thyroid stimulating hormone (TSH), lipid profile, and von Willebrand’s profile. Patients with scores either < 50 or > 150 IU were classified as abnormal for von Willebrand’s antigen. Dysregulation of simultaneously measured adrenocorticotropic hormone (ACTH)/cortisol and antidiuretic hormone (ADH)/osmolality was determined by (i) absolute high (ACTH > 45 or cortisol > 21; ADH > 13 or osmolality > 300) or low (ACTH < 5 or cortisol < 4; ADH < 1.3 or osmolality < 275) values; or (ii) ACTH was below 10 when cortisol was below 7; or ADH was below 2.2 when osmolality was 292–300 for the two-paired tests; or (iii) ACTH was > 15 when cortisol was > 16; and ADH > 4.0 when osmolality was 275–278 for the two-paired tests.

### RNA collection, extraction and labeling

Venous blood was drawn from the arms of subjects into PAXgene RNA blood collection tubes (http://www.preanalytix.com/product-catalog/blood/rna/products/paxgene-blood-rna-tube/), incubated for six hours at room temperature, then frozen at −80°C until RNA extractions were performed. Total RNA was extracted with the Qiagen PAXgene Blood miRNA System kit according to manufacturer’s protocol. The total RNA was analyzed using an Agilent 2100 bioanalyzer for RNA integrity (only using samples with RIN scores ≥ 8) and was then quantified using a NanoDrop ND-1000 (Wilmington, DE).

Fluorescent labeling of RNA was performed according to the manufacturer’s instructions for the Agilent Quick Amp labeling kit. Total RNA (100 ng) was amplified and labeled with Cy3 dye. This amplification product was measured for quantity and dye incorporation using the Nanodrop ND-1000 and then hybridized to Agilent catalogue Human Whole Genome V2 microarrays.

### Microarray hybridizations and scanning

All microarray hybridizations were performed according to the manufacturer’s instructions in the One-Color Microarray-Based Gene Expression Analysis manuals with only one modification. Instead of hybridizing the recommended amount of 1.6 μg Cy-3 labelled RNA to the array, we used 2.4 μg to boost the signal because the hemoglobin RNA removal step was not employed (this increase was used only after careful analysis of its reproducibility and results as compared with the standard protocol). The fluorescently labelled RNA was hybridized to the microarray at 65 °C in a rotating oven. After 17 hours, the arrays were washed consecutively in solutions of 6X SSPE with 0.005% N-lauroylsarcosine and 0.06X SSPE with 0.005% N-lauroylsarcosine for 1 min each at room temperature. This wash was followed by a final 15 s wash in acetonitrile. Microarrays were then imaged on an Agilent microarray scanner and extracted using Agilent Feature Extraction 9.5.

### Microarray analysis

All scan data were imported into GeneSpring Multi-Omic analysis software version 12.6.1 and normalized using the default parameter of the 75^th^ percentile shift. Probes were first filtered by intensity, only keeping ones with a minimum of 40 counts in at least 50% of samples that constituted either the patient or control class. Probe intensity values for each class were averaged and subjected to a moderated *t*-test. By using the moderated *t*-test p value (p) and patient vs control fold change (FC) cut-offs, two gene lists of interest were then generated with different levels of stringency. The low stringency (LS) gene set contained probes with a p < 0.05 and a FC > 1.4, while the high stringency gene set (HS) contained probes with a p < 0.01 and FC > 1.5. Each gene set was subjected to gene ontology (GO) and KEGG molecular pathway analysis to identify the possible enrichment of genes with specific biological themes using the web tool Database for Annotation, Visualization and Integrated Discovery (DAVID) 6.7.

Data was then analyzed in two ways, first by using all profiles individually, and second by averaging the replicate arrays for each subject, with the averaged replicate data results reported in Supplementary material. Gene set data were subjected to Principal Component Analysis (PCA) as well as classification algorithms, such as Support Vector Machines (SVM), within the GeneSpring environment to determine if differences in gene expression could be used to stratify patient from control samples.

### qPCR

Eight genes selected from the array data were analyzed by quantitative real-time PCR (qPCR) in seven patients and seven controls (these subjects were selected based on quantity of RNA remaining after microarrays). Sequences of hydatidiform mole associated and imprinted (non-protein coding) (HYMAI, access number: NR_002768), von Willebrand factor (VWF, NM_000552), coagulation factor XIII, A1 polypeptide (F13A1, NM_000129), interleukin 18 receptor accessory protein (IL18RAP, NM_003853) and T cell receptor beta variable 28 (TCRb28, AB305916) were selected for primer design using PrimerQuest (https://www.idtdna.com/Scitools/Applications/Primerquest/). Primers for CD9 molecule (CD9), chromosome X open reading frame 65 (CXorf65) and coiled-coil domain containing 12 (CCDC12) were selected from PrimerBank (http://pga.mgh.harvard.edu/primerbank/). CCDC12 was used a reference gene as its expression was unchanged and stable for the subjects in this cohort. Primers were synthesized by Integrated DNA Technologies (Coralville, IA) and specificities were verified by BLASTN analysis against all GenBank entries. Relative levels of mRNA were determined using qPCR on an ABI7000 (Applied Biosystems). Optimized qPCR parameters for each gene were determined using pooled cDNA (1:10 diluted), reverse transcribed from 1200 ng of total RNA from two control subjects with a high capacity RNA-to-cDNA Kit (Applied Biosystems, Foster, CA) according to manufacturer’s instructions. Reactions were performed in a volume of 20 μl with primer concentration of 300 nM, 10 μl SYBR Green Master Mix (Applied Biosystems) and 2 μl 1:10 diluted cDNA. At the end of each real-time PCR reaction, PCR products were subjected to a melt curve analysis. Negative controls (minus reverse transcriptase) were run for each gene to confirm the absence of contaminating DNA. Amplification efficiencies (E) were estimated from a six-point standard curve, ranging from 1:10 to 1:5000 dilution, for each gene using the formula E = 10^-1/slope^-1 [14] resulting in a correlation coefficient R^2^ > 99.4% and E = 0.976–1.045. All samples and negative controls were reverse transcribed in duplicate. qPCR for each gene was then run in duplicate from each duplicate RT for a total of four values for each subject/gene pair. These four Ct values for each subject/gene were averaged and then normalized to the corresponding measured mRNA Ct value of the reference gene CCDC12, which did not show significant variation across samples in microarray hybridizations. Comparative Ct method of analysis (2^-ΔΔCt^) was used to determine changes in gene expression between patients and normal controls.

## Results

### Microarray

All microarray data have been uploaded to NCBI’s Gene Expression Omnibus (GEO, http://www.ncbi.nlm.nih.gov/geo/, GEO series accession number GSE58625) for independent analysis. Each array passed all quality control standards as recommended by the manufacturer, Agilent Technologies. Duplicate (technical replicate) whole genome microarrays for 11 patients (9 males and 2 females; mean age 51.4, SD 6.1) and 11 controls (9 males and 2 females; mean age 50.7, SD 7.8), for a total of 44 arrays, were uploaded to GeneSpring. The arrays were divided into patient and control classes, and the probes were filtered for signal intensities of greater than 40 in at least 50% of the samples in one of the two classes. This reduced the number of experimental probes from 34,183 to 19,495. The data were then subjected to a moderated *t*-test and filtered by p-values and FCs to produce two gene sets, LS and HS. The LS filtering resulted in 182 significant probes (Additional file 3: Table S3) while the HS filtering resulted in 55 probes (Table 1). The FC cut-off is considered the best indicator of true differential gene expression [15,16] and qPCR validation of Agilent microarray results at a minimum threshold of 1.4 FC has been robust at this specific genomic core [17]. Most differential gene expression remained under a 2 fold change, although the little studied sialic acid-binding immunoglobulin-type lectin, SIGLEC14 exhibited by far the greatest degree of change at 7.5 FC. However, that large change was due to the fact that 3 of the 11 controls expressed the gene at undetectable levels, while all patients had detectable levels of expression.

**Table 1:** **HS gene list**

**p value**	**FC**	**GeneSymbol**	**Description**	**Genbank Acc**
3.36E-04	1.56	ATHL1	Acid trehalase-like 1	NM_025092
5.53E-03	-1.61	APCDD1	Adenomatosis polyposis coli down-regulated 1	NM_153000
2.02E-04	1.63	AEBP1	AE binding protein 1	NM_001129
1.47E-03	1.52	ABCB1	ATP-binding cassette, sub-family B (MDR/TAP), member 1	NM_000927
2.91E-05	1.62	*CELSR3*	*Cadherin, EGF LAG seven-pass G-type receptor 3*	NM_001407
1.34E-03	1.62	CD274	CD274 molecule (CD274), transcript variant 1	NM_014143
7.44E-05	-1.56	CD36	CD36 molecule (thrombospondin receptor)	NM_001001547
4.01E-04	-1.90	**CD9**	**CD9 molecule**	NM_001769
9.33E-05	1.51	*LOC100289090*	*cDNA FLJ33063 fis, clone TRACH2000047*	AK057625
3.24E-04	1.60	LOC692247	cDNA FLJ36520 fis, clone TRACH2002100	AK093839
4.36E-03	1.51	C17orf66	Chromosome 17 open reading frame 66	NM_152781
4.00E-06	1.81	C6orf163	Chromosome 6 open reading frame 163	NM_001010868
7.42E-04	-1.68	CYB5R2	Cytochrome b5 reductase 2	NM_016229
1.33E-03	2.16	DDX43	DEAD (Asp-Glu-Ala-Asp) box polypeptide 43	NM_018665
7.91E-03	1.76	EIF4G3	Eukaryotic translation initiation factor 4 gamma 3	NC_018912.2
3.33E-07	1.51	*N/A*	*Formin-like 1 protein (Leukocyte formin)*	AC_000149.1
4.71E-04	1.67	GPR125	G protein-coupled receptor 125	NM_145290
8.75E-03	1.79	GATA2	GATA binding protein 2 (GATA2), transcript variant 1	NM_001145661
5.97E-03	1.54	GZMK	Granzyme K (granzyme 3; tryptase II)	NM_002104
3.89E-04	1.51	GBP5	Guanylate binding protein 5 variant 1	NM_052942
1.74E-03	1.53	LRRC37A4P	Hypothetical protein LOC652203	BC107850
1.01E-03	-3.85	ITGB2	Integrin beta chain, beta 2 variant (Fragment)	NC_018932.2
5.33E-03	-1.69	IL5RA	Interleukin 5 receptor, alpha, variant 3	NM_175725
1.75E-03	1.66	NEAT1	lncRNA Nuclear paraspeckle assembly transcript 1	AF001893
6.55E-04	1.55	LINC00282	lncRNA 282 (LINC00282)	NR_027047
1.08E-04	1.58	**HYMAI**	**lncRNA Hydatidiform mole associated and imprinted**	NR_002768
3.66E-04	1.74	LMF1	lncRNA Lipase maturation factor 1 variant 4	NR_036442
3.62E-05	-1.89	N/A	lncRNA TCONS_l2_00011463	NC_018928
4.20E-03	-1.73	LYPD2	LY6/PLAUR domain containing 2	NM_205545
4.37E-03	-1.63	MARCO	Macrophage receptor with collagenous structure	NM_006770
1.85E-03	-2.43	N/A	Major histocompatibility complex, class I, V (pseudogene)	NC_018917
2.95E-03	-1.61	HLA-DQB1	MHC class II, DQ beta 1 variant 1	NM_002123
6.20E-03	-2.16	HLA-DQB1	MHC class II, DQ beta 1 variant 2	NM_001243961
8.09E-04	-1.54	HLA-DQB1	MHC class II, DQ beta 1 variant 3	NM_001243962
2.96E-04	-2.13	PADI6	Peptidyl arginine deiminase, type VI	NM_207421
4.12E-05	-2.26	PEX6	Peroxisomal biogenesis factor 6	NM_000287
3.69E-04	-1.82	PEX6	Peroxisomal biogenesis factor 6	NM_000287
9.37E-03	1.55	PDE4DIP	Phosphodiesterase 4D interacting protein variant 1	NM_014644
3.56E-03	1.80	PLGLB1	Plasminogen-like B1	BC022294
2.19E-03	1.51	LOC345051	Predicted: hCG38984 variant X1	XR_109844
3.22E-03	-1.74	PPP1R17	Protein phosphatase 1, regulatory subunit 17 variant 1	NM_006658
8.37E-03	-1.67	PDK4	Pyruvate dehydrogenase kinase, isozyme 4	NM_002612
2.41E-04	-1.51	ARHGAP22	Rho GTPase activating protein 22, variant 3	NM_021226
8.34E-04	1.52	ARHGEF1	Rho guanine nucleotide exchange factor (GEF) 1	AK090448
6.37E-03	7.57	SIGLEC14	Sialic acid binding Ig-like lectin 14	NM_001098612
8.88E-06	1.55	*SIGIRR*	*Single immunoglobulin and toll-interleukin 1 receptor*	XM_005253044
1.58E-04	1.53	*TCRBV27*	*T cell receptor beta variable 27*	AB305921
1.22E-03	2.02	**TCRBV28**	**T cell receptor beta variable 28**	AY751906
1.55E-03	2.34	**TCRBV28**	**T cell receptor beta variable 28**	AB305916
3.33E-04	1.51	TCRBV6	T cell receptor beta variable 6-4	A25966
6.60E-03	-1.77	TOLLIP	Toll interacting protein	NM_019009
3.79E-03	3.03	TUBB2A	Tubulin, beta 2A class Iia	NM_001069
1.36E-03	1.55	VIPR2	Vasoactive intestinal peptide receptor 2	NM_003382
3.82E-03	-1.71	**VWF**	**von Willebrand factor**	NM_000552
8.82E-03	2.74	ZFP57	ZFP57 zinc finger protein	NM_001109809

Genes that exhibited a FC > 1.5 and p < 0.01 in patients v controls. **Bold** shading indicates PCR analysis, *italics* indicates genes with the highest loading factors in PCA analysis. Genes sorted by description.

The LS and HS gene sets were subjected to gene ontology analysis using both GeneSpring and the web tool DAVID 6.7 [18]. Although no significant results were generated after Benjamini multiple test corrections were applied, the top hits in the biological process category were (1) wound healing with an uncorrected p-value of 0.00016 (corrected p = 0.0899), (2) platelet alpha granule with an uncorrected p-value of 0.00069 (corrected p = 0.109) and (4) coagulation with an uncorrected p-value of .0044 (corrected p = 0.359). The genes CD9, CD36, VWF and Factor XIII were included in all three categories.

To determine the power of the experiment, an online calculator at the U. of Texas MD Anderson bioinformatics website (http://bioinformatics.mdanderson.org/MicroarraySampleSize/) was used. Because the sample size of 11 is small, the estimated power of the experiment was found to be 0.6, meaning that only an estimated 60% of true differentially expressed genes will be identified. In an effort to further validate genes of interest, we compared the 11 patients to a wider control database run on the same platform. This larger control data set was made up of 79 gene expression profiles from 30 male and 18 female control subjects between the ages of 25 and 65 (48.7 mean). Additional file 4: Figure S1 shows 21 box whisker plots of genes of interests found throughout the discussion.

### PCR

All PCR primer pairs (Additional file 5: Table S4) were optimized to produce single band products when run on the Agilent Bioanalzyer 2100. Average Cts for all subjects in this cohort were as follows, VWF = 28.19, HYMAI = 27.8, CXorf65 = 25.9, CD9 = 25.4, TCRb28 = 24.3, F13A1 = 23.6, IL18RAP = 23.5 and the reference gene CCDC12 = 24.5.

**Figure 1:**
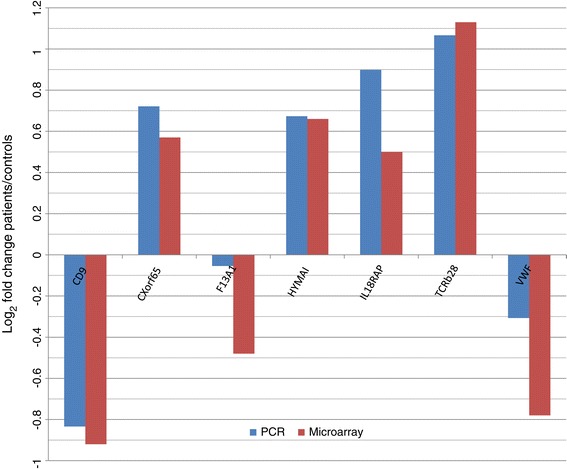
**PCR validation of microarray.** PCR results were compared with microarray data. Pearson correlation between the two methods was 0.955, with a p = 0.00076.

The PCR results were strongly supportive of the microarray data (Figure 1) with a Pearson correlation coefficient of 0.955 (p = 0.00076) across the seven genes analyzed. When the Ct values for patients and controls were compared by *s*-test, significant differences were shown for HYMAI (p = 0.03), CD9 (p = 0.006), CXorf65 (p = 0.05), IL18RAP (p = 0.005) and TCRb28 (p = 0.02). However, no significant differences were found between controls and patients for VWF (p = 0.40) and F13A1 (p = 0.87) as the PCR results, although corroborating the down-regulation of both genes in patients, showed a smaller change than the microarray.

### PCA plot and SVM classification

**Figure 2:**
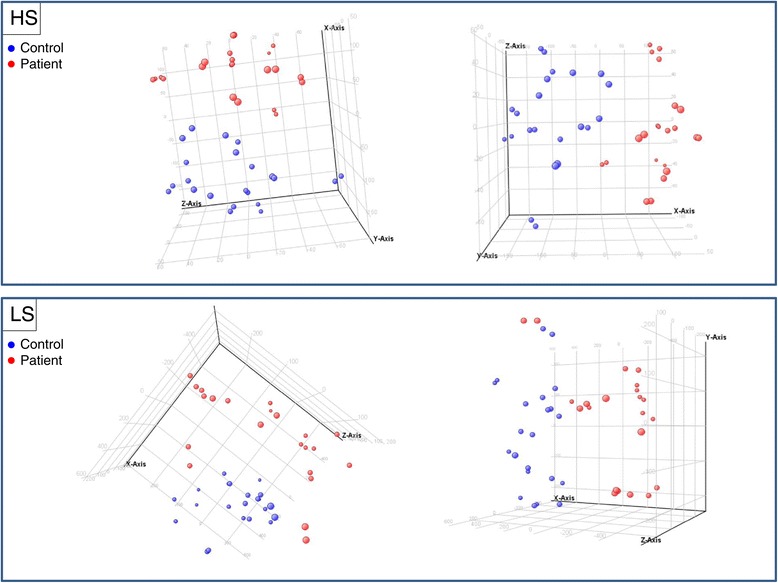
**PCA plot.** Control and patient profiles were subjected to principal component analysis and rotated to angles that visually best exhibit their separation. X, Y and Z axes indicate first three principle components. HS = high stringency, LS = low stringency gene sets.

Using the HS and LS gene sets, a PCA was performed, and an SVM classification algorithm was run inside the GeneSpring environment. The PCA plot produced a clear separation between control and patient samples (Figure 2 and Additional file 6: Figure S2). However, the smaller HS set provided a tighter grouping of samples. Genes with the highest loading factors were TCRBV27, SIGIRR, CESLR3, cDNA FLJ33063 and formin like protein, and are highlighted in Table 1. It was typical that the top and bottom 10 values of the 44 total data profiles were exclusively controls and cases, respectively, for each of these genes. For example, of the 44 normalized values for the gene SIGIRR (up in patients 1.7 fold), the lowest 8 values were all control with the 9^th^ lowest being a patient, while the 12 highest values were patients with the 13^th^ highest being a control.

Using a polynomial kernel and a “leave one out” training method, an SVM classification algorithm was built by further removing 2 randomly selected male control and patient data. Eight profiles from 4 subjects (i.e., both replicates) were removed from the pool, leaving 36 out of 44 data profiles for training. The algorithm correctly classified patient and control samples with 100% accuracy during training for both the HS and LS gene sets. When the prediction model was run using all 44 profiles it still correctly predicted the classes of all the samples for both averaged and individual replicates. Table 2 (and Additional file 7: Table S5) shows the results of analysis with the HS data set, and although three of the four unknown sample predictions were of the lowest confidence for the entire group, one of the unknown samples showed a classification confidence of nearly 100%.

**Table 2:** **SVM classification**

**Identifier**	**Gender**	**Trained**	**Predicted**	**Confidence**
**Control 1-1**	Female	Control	Control	0.754
**Control 1-2**	Female	Control	Control	0.764
**Control 2-1**	Male	Control	Control	0.796
**Control 2-2**	Male	Control	Control	0.789
**Control 3-1**	Male	Control	Control	0.794
**Control 3-2**	Male	Control	Control	0.829
**Control 4-1**	Male	Control	Control	0.805
**Control 4-2**	Male	Control	Control	0.832
**Control 5-1**	Male	Control	Control	1.000
**Control 5-2**	Male	Control	Control	1.000
**Control 6-1**	Male	Control	Control	0.826
**Control 6-2**	Male	Control	Control	0.821
**Control 7-1**	Female	Control	Control	0.877
**Control 7-2**	Female	Control	Control	0.827
**Control 8-1**	Male	Control	Control	0.710
**Control 8-2**	Male	Control	Control	0.772
**Control 9-1**	Male	Control	Control	0.796
**Control 9-2**	Male	Control	Control	0.936
**Patient 1-1**	Male	Patient	Patient	0.819
**Patient 1-2**	Male	Patient	Patient	0.805
**Patient 2-1**	Male	Patient	Patient	0.858
**Patient 2-2**	Male	Patient	Patient	0.847
**Patient 3-1**	Male	Patient	Patient	0.858
**Patient 3-2**	Male	Patient	Patient	0.917
**Patient 4-1**	Female	Patient	Patient	0.858
**Patient 4-2**	Female	Patient	Patient	0.853
**Patient 5-1**	Male	Patient	Patient	0.844
**Patient 5-2**	Male	Patient	Patient	0.813
**Patient 6-1**	Male	Patient	Patient	0.929
**Patient 6-2**	Male	Patient	Patient	0.898
**Patient 7-1**	Male	Patient	Patient	0.763
**Patient 7-2**	Male	Patient	Patient	0.852
**Patient 8-1**	Male	Patient	Patient	0.790
**Patient 8-2**	Male	Patient	Patient	0.821
**Patient 9-1**	Female	Patient	Patient	0.847
**Patient 9-2**	Female	Patient	Patient	0.904
*Control 10-1	Male		Control	0.449
*Control 10-2	Male		Control	0.399
*Control 11-1	Male		Control	0.627
*Control 11-2	Male		Control	0.660
*Patient 10-1	Male		Patient	0.019
*Patient 10-2	Male		Patient	0.108
*Patient 11-1	Male		Patient	0.976
*Patient 11-2	Male		Patient	1.000

Results of predictions and confidence for cases of CIRS-ciguatera and controls using a Support Vector Machines classification algorithm, while withholding eight random data profiles* from training.

## Discussion

Although many important genes may remain undetected or overlooked, we have endeavored to highlight several pathways, as well as individual genes, that likely contribute to CIRS-ciguatera using data predominantly from our HS results of 55 probes.

### Coagulopathies and hemostasis

Previous functional genomics studies in mice have shown disruption in the complement and coagulation pathways after an acute exposure to ciguatoxin [9]. Additionally, human cases of CIRS-ciguatera have been identified with similar abnormalities, such as elevated complement C4a, irregularities in VWF and Factor VIII, and elevated levels of plasminogen activator inhibitor-1 [5]. The HS gene set identified in the current study contains VWF, coagulation factor XIII, thrombospondin (CD36) and CD9. These genes function in platelet activation, which was one of the most enriched pathways revealed by DAVID GO analysis. CD9 is a member of the tetraspanin family and one of the most abundant proteins on the platelet cell membrane [19]. It was determined to be down-regulated here by microarray (1.9 fold) and validated by qPCR (1.8 fold), and it has long been known to play a role in cell motility and adhesion [20]. An interesting aspect of CD9 in the case of CIRS-ciguatera is that it influences the expression of MMP9, which is found up-regulated at the protein level in the plasma of over 60% of CIRS-ciguatera cases. In keratinocytes, down-regulation of CD9 was found to increase activity and expression of MMP9 through JNK signaling [20], while in a fibrosarcoma cell line, epithelial growth factor receptor, EGFR, was an important intermediary for increased release of pro-MMP9 by CD9 [21]. CD9 is critical for platelet microparticle release, which are formed from budding of the cytoplasmic membrane and are an order of magnitude larger than exosomes [19]. Roughly 70–90% of all microparticles in the bloodstream originate from platelets [22]. They are thought to be part of a wide-ranging form of inter-cellular communication that function by carrying bioactive components and signaling molecules, which can be released into target cells, in many instances, altering the function of those cells [22]. In addition to their function in coagulation, the microparticles are also implicated in a range of inflammatory and autoimmune diseases [22,23]. A recent study identified another high density platelet receptor, CD36 (aka, thrombospondin receptor), which was similarly down-regulated in these CIRS-ciguatera patients (1.56 fold), that strongly co-localized with CD9, not only on the surface of platelets, but also on dermal microvessel endothelial cells, possibly forming sites for platelet adhesion and aggregation [24]. These receptors, together with the genes for VWF and Factor XIII, which were also down-regulated in this study, most likely play a role in the coagulopathies and hemodynamics associated with CIRS-ciguatera. What is also interesting is that one of the most common Folk tests for ciguatoxic fish used in the South Pacific is called the bleeder test. This test relies on hemorrhaging of an incision in the tail of the fish to indicate toxicity [25].

### Long non-coding RNA (lnc-RNA)

Several lncRNAs were found to be differentially expressed in this study, leading us to believe that these regulators of transcription and translation play an important role in CIRS-ciguatera. Non-coding RNA (ncRNA) research is a relatively new field and, although microRNA research far outpaces lncRNA, its importance is becoming noteworthy as some single lncRNA knockouts have proven lethal in mice [26]. Relatively little is known about the functions of most lncRNAs; however, a few species have been characterized because of the significance of their functions. One such species was found to be up-regulated 1.66 fold in CIRS-ciguatera patients, Nuclear Enriched Abundant Transcript 1, NEAT1. NEAT1 is a critical component of nuclear paraspeckles, a nuclear body found to have a role in sequestering certain RNA transcripts in the nucleoplasm, thus keeping them from being translated into proteins in the cytoplasm. It is thought that this complex binds transcripts through their edited 3′ UTR that is then cleaved under a stress response, which allows the transcripts to be sent immediately to the cytoplasm for translation [27]. In a different paradigm of transcriptional control, up-regulation of NEAT1 was described in response to viral infection and Toll receptor pathways, which in turn led to increased binding and sequestration of IL-8 transcriptional repressors by the paraspeckle, thus allowing increased expression of the cytokine [28]. Early work identified a smaller form of NEAT1 in trophoblasts that could suppress gene expression of the major histocompatability complex (MHC) through sequestration of a critical transcription factor, signal transducer and activator of transcription 1 (STAT1), although in this instance, STAT1 was sequestered in the cytoplasm instead of the nucleus [29]. This fetal isoform of NEAT1 was not found expressed in adult tissues, which is interesting because several MHC genes were significantly down-regulated in our patient samples while STAT1 was significantly up-regulated 1.42 fold.

### MHC and adaptive immunity

Adaptive immunity involves the destruction of foreign pathogens and presentation of their peptide remains to T cells to begin the processes of antibody production by B cells, and “attack on site” natural killer (NK) cells and cytotoxic T cells. The direct killing of cells usually involves the emptying of lytic enzymes, granzymes (GZM), onto targeted cells. Until recently, it was thought that these cells acted exclusively as part of the innate immune response. However, new evidence has shown that these cells also shape adaptive responses and can play a role in autoimmune disorders, such as Multiple Sclerosis, where CD56^bright^ NK cells were shown to kill activated autologous T cells through GZM-K (up-regulated 1.54 fold here) dependent mechanisms. GZMK has also been suggested as a marker for different stages of sepsis [30]. The heterodimeric HLA-DQ protein, composed of an alpha and beta subunit, acts as an antigen-presenting molecule of foreign peptide fragments, as opposed to MHC class I proteins that present self molecules. Several probes for the MHC class II gene HLA-DQB were found down-regulated in CIRS-ciguatera patients. In earlier work, we discovered that certain DRB and DQB class II HLA haplotypes were more susceptible to chronic illness caused by acute exposure to ciguatoxins [5]. This is not unusual in chronic inflammatory/autoimmune illnesses. In fact, the greatest risk factor for Type I diabetes in Caucasians and African Americans was found to be certain HLA-DQ genotypes [31], as was DRB-DQ genotypes in late autoimmune diabetes in adults (LADA) [32]. Additionally, two DQ haplotypes are linked with nearly all celiac disease (CD) [33], the DQ2 haplotype, which is present in more than 95% of CD, and DQ8, which is present in the remainder. These two haplotypes are also over-represented in many other inflammatory disorders, including Addison’s disease, Hashimotos thyroiditis, Graves’ disease, Sjogren’s syndrome and others (for review see [33]).

Antigen presenting cells (APC) display foreign peptide fragments through MHC receptors, these fragments are then probed by T cells through the T cell receptor (TCR). The TCR is a heterodimer, typically composed of alpha and beta subunits, with each subunit containing a variable region that interacts with the APC presented peptide. Out of a possible 46 TCR beta variable (TCRBV) probes (coding for 45 genes), our LS set of 193 significant probes contained six, all up-regulated from 1.51 to 2.33 FC. T cells not only combat infectious pathogens, they are also important in the surveillance of autoreactive self antigens that can result in autoimmune syndromes or cancer. The gene CD274, aka programmed cell death ligand 1 (PDL1, or B7-H1), up-regulated 1.7 fold here, is important for maintaining peripheral tolerance and is a powerful suppressor of T cell-mediated immunity. Over expression of CD274 by tumors, or due to chronic infection allows immune avoidance through T cell anergy, exhaustion, apoptosis and other mechanisms, and high levels of the receptor have been correlated with poor prognoses for certain cancers [29]. The up-regulation of TCRs and PDL1 seem to indicate an attempt to generate T cells clones for a specific antigen.

### Inflammation

Our findings are replete with genes involved in inflammation, and although a simple explanation of how this differential expression accounts for CIRS is impossible, there are too many important inflammatory threads to ignore. Activation of inflammation is well documented through Toll-like receptors (TLR) and Interleukin-1 receptor like receptor (ILR) signaling. The single immunoglobulin and IL-1 receptor (SIGIRR), found up-regulated in our patients 1.55 fold, is implicated in infectious and sterile inflammation, as well as autoimmune disease. It is also known to blunt the signaling of both TLR and IL-1, in addition to other interleukins such as IL-18 (for review see [34]). The IL-18 receptor is an alpha-beta heterodimer, and the beta subunit, IL-18RAP, was up-regulated in patients 1.4 fold. This subunit is also part of a linkage disequilibrium block found in CD, and its mRNA expression in blood is an important disease correlate [35]. VIP was shown to down-regulate TLRs while at the same time up-regulating SIGIRR expression [36]. Since over 90% of CIRS-ciguatera patients have findings of abnormally low VIP in plasma [5], the down-regulation of the VIP receptor found here, VIPR2 (1.7 fold), would seemingly further diminish the anti-inflammatory effects of what little neuropeptide is circulating in the blood. Toll interacting protein (TOLLIP), down-regulated in patients 1.77 fold, is an inhibitor of TLR signaling and has also been shown to modulate both IL-1 and LPS inflammatory pathways, as well as TNFα and IL-6 gene expression [37]. Its role as an immune regulator was further expanded when it was found to inhibit TGFβ signaling [38], which could prove important in CIRS-ciguatera because TGFβ-1 is abnormally high in over 90% of cases.

### Proteomics

Because ciguatoxins are toxic in minute amounts, their detection in tainted fish or exposed patients is extremely difficult, and the diagnosis of CFP relies predominantly on symptoms following a fish meal. Abnormalities in blood proteomic measurements of MSH, VIP, C4a, TGFβ-1, MMP9, ADH/osmolality, ACTH/cortisol and/or VEGF are routinely found in the chronic syndrome, but we do not know if these anomalies are detectable at the acute stage. Additionally, these proteomic abnormalities are not unique to CFP, but are also found in patients with other biotoxin-induced CIRS such as toxic mold exposure [39] and acute Lyme disease [40]. The concept of a “final common pathway” of chronic immune activation seen in these diverse syndromes is consistent with the presentation of a multisystem illness that is not defined by any single symptom or group of symptoms, or unique proteome with our current testing. This increases the importance of identifying a genomic fingerprint for these different illnesses to provide objective support for use in differential diagnosis. Additionally, since the proteome of whole blood is not simply the product of white blood cells, but is more so the product of all the organs being supplied, there is no tidy explanation to correlate gene expression with the proteomic abnormalities we have recorded. However, the benefit of this paradigm in whole blood is that while the genomic research interrogates the leukocytes, the proteomics can interrogate the entire body. Further research is needed to clarify this dichotomy of genomic responses in white cells separately from the proteome of blood in inflammatory illnesses.

## Conclusions

Symptoms of the acute stage of ciguatera are easily explained by circulating toxin, but chronic illness is not. We cannot say that all chronic illness after ciguatoxin exposure results in a CIRS syndrome, but of the cases we see, an immune disequilibrium after the acute trigger is quite typical. The role of platelet microparticles has not been defined in any of the CIRS illnesses to date, but with growing evidence of their potential pathology and importance in communication and direction of immune functions, we will include their study going forward. Dysregulation of the HLA/T-cell receptor axis seen here reminds us of autoimmune elements seen in other inflammatory illnesses, such as CD. With susceptible HLA haplotypes recorded previously in CIRS-ciguatera, also similar to CD, as well as IgGs reactive to gliadins, gluten produced inflammation could be an important contributor to a protracted recovery in these patients, as could many other inflammagens imposing on a hyper-active immune system. By elucidating the roles of environmental triggers and predisposing genetic backgrounds, the etiology of inflammatory illnesses is becoming more transparent. Now, with several innate and adaptive inflammatory genes to investigate further, we feel that genomic testing will serve an expanded clinical role as work proceeds on defining the parameters of acute ciguatera and CIRS.

## Additional files

Additional file 1: Table S1. Acquisition, duration and fish species. Geographic location of exposure, duration of illness at time of sampling and fish consumed in cases of ciguatera CIRS. Additional file 2: Table S2. Symptoms. Number and type of symptoms reported by 11 ciguatera fish poisoning patients prior to, and during, acute and chronic phases of exposure. Additional file 3: Table S3. LS Gene List. Probes can be identified by Agilent probe ID in cases where GenBank accession is not available. Additional file 4: Figure S1. Gene Expression Analysis with Extended Control Database. Box whisker plots show specific gene expression comparisons between these 11 CIRS ciguatera patients with 79 gene expression profiles from 30 male and 18 female control subjects. The shaded box indicates the interquartile range of values (25% - 75%) while the interior solid line indicates the median. Additional file 5: Table S4. Primers used in qPCR reactions. PB: PrimerBank: http://pga.mgh.harvard.edu/primerbank/. * denotes transcript used as a reference. Additional file 6: Figure S2. PCA of averaged replicates. Replicate control and patient profiles were averaged then subjected to principal component analysis. Plots were rotated to angles that visually best exhibit their separation. X, Y and Z axes indicate first three principle components. HS = high stringency, LS = low stringency gene sets. Additional file 7: Table S5. SVM Classification of averaged replicates. Results of predictions and confidence for the average of duplicate arrays for cases of CIRS-ciguatera and controls using a Support Vector Machines classification algorithm, while withholding four data profiles* from training.
