# Supplementary material for: Transcriptomic signatures in whole blood of patients who acquire a chronic inflammatory response syndrome (CIRS) following an exposure to the marine toxin ciguatoxin
Source: BMC Med Genomics. 2015 Apr 2;8:15. doi: 10.1186/s12920-015-0089-x (PMC4392619; doi:10.1186/s12920-015-0089-x)
Supplement: Additional file 1: Table S1. — Acquisition, duration and fish species. Geographic location of exposure, duration of illness at time of sampling and fish consumed in cases of ciguatera CIRS. [file 12920_2015_89_MOESM1_ESM.pdf]

Site of acquisition	Duration	Species implicated
China	1 year	3 different species in a single meal
St. John	1 year	Barracuda
Yucatan	3 years	Snapper
Fiji	3 years	Barracuda
Fiji	3 years	Barracuda
Hawaii	4 years	Roi (peacock grouper)
Caribbean	4 years	Grouper
Caribbean	5 years	Grouper
Caribbean	6 years	Grouper
Panama	7 years	Jack
Miami	8 years	Jack

Supplementary table 1. Acquistion, duration and fish species. Geographic location of exposure, duration of illness at time of sampling and fish consumed in cases of ciguatera CIRS
