# Supplementary material for: Transcriptomic signatures in whole blood of patients who acquire a chronic inflammatory response syndrome (CIRS) following an exposure to the marine toxin ciguatoxin
Source: BMC Med Genomics. 2015 Apr 2;8:15. doi: 10.1186/s12920-015-0089-x (PMC4392619; doi:10.1186/s12920-015-0089-x)
Supplement: Additional file 2: Table S2. — Symptoms. Number and type of symptoms reported by 11 ciguatera fish poisoning patients prior to, and during, acute and chronic phases of exposure. [file 12920_2015_89_MOESM2_ESM.pdf]

Category	Pre-exposure	Acute	Chronic
Fatigue	2	6	10
Weakness	1	5	11
Ache	2	4	9
Cramp	0	3	8
Unusual pain	0	2	6
Ice pick pain	0	1	4
Headache	1	6	7
Light sensitivity	0	3	10
Red eyes	1	2	8
Blurred vision	0	2	9
Tearing	0	2	3
Sinus	1	2	6
Cough	0	0	1
Shortness of breath	0	2	7
Abdominal pain	1	8	8
Diarrhea	1	10	7
Joint Pain	1	2	7
Morning stiffness	0	2	6
Memory	0	5	10
Focus/concentration	0	5	11
Word recall	0	4	10
Information assimilation	0	4	10
Confusion	0	3	10
Disorientation	0	3	6
Itching	0	5	8
Mood swings	0	2	9
Appetite	0	0	6
Sweats	0	8	4
Temp regulation	0	3	8
Thirst	0	0	5
Increased urination	0	2	8
Static shocks	0	0	2
Numbness	0	4	9
Tingling	0	6	9
Vertigo	0	4	6
Metallic taste	1	6	6
Tremor	0	4	8
Hot/cold reversal	0	8	4

Supplementary Table 2. Symptoms. Number and type of symptoms reported by 11 ciguatera fish poisoning patients prior to, and during, acute and chronic phases of exposure.
