# Supplementary material for: Transcriptomic signatures in whole blood of patients who acquire a chronic inflammatory response syndrome (CIRS) following an exposure to the marine toxin ciguatoxin
Source: BMC Med Genomics. 2015 Apr 2;8:15. doi: 10.1186/s12920-015-0089-x (PMC4392619; doi:10.1186/s12920-015-0089-x)
Supplement: Additional file 3: Table S3. — LS Gene List. Probes can be identified by Agilent probe ID in cases where GenBank accession is not available. [file 12920_2015_89_MOESM3_ESM.pdf]

Agilent Probe	p value	FC	GeneSymbol	Description	GenbankAccession	EntrezGeneID
A_23_P49924	4.58E-03	1.43	NT5C3B	5'-nucleotidase, cytosolic IIIB (NT5C3B), transcript variant 1, mRNA [NM_052935]	NM_052935	115024
A_23_P148194	3.70E-05	-1.41	ADI1	acireductone dioxygenase 1 (ADI1), mRNA [NM_018269]	NM_018269	55256
A_23_P337262	5.53E-03	-1.61	APCDD1	adenomatosis polyposis coli down-regulated 1 (APCDD1), mRNA [NM_153000]	NM_153000	147495
A_23_P137931	1.09E-02	-1.44	ADORA3	adenosine A3 receptor (ADORA3), transcript variant 2, mRNA [NM_000677]	NM_000677	140
A_33_P3396010	8.58E-04	1.49	AGER	advanced glycosylation end product-specific receptor (AGER), transcript variant 9, mRNA [NM_001206966]	NM_001206966	177
A_23_P157299	2.02E-04	1.63	AEBP1	AE binding protein 1 (AEBP1), mRNA [NM_001129]	NM_001129	165
A_33_P3380311	3.96E-02	1.50	ATRX	alpha thalassemia/mental retardation syndrome X-linked (ATRX), transcript variant 1, mRNA [NM_000489]	NM_000489	546
A_24_P174503	2.41E-04	1.45	AMT	aminomethyltransferase (AMT), transcript variant 1, mRNA [NM_000481]	NM_000481	275
A_33_P3245449	1.98E-02	-1.63	LOC389834	ankyrin repeat domain 57 pseudogene (LOC389834), non-coding RNA [NR_027420]	NR_027420	389834
A_33_P3263666	1.42E-02	-1.50	ANKRD9	ankyrin repeat domain 9 (ANKRD9), mRNA [NM_152326]	NM_152326	122416
A_32_P148345	2.97E-02	-1.49	ANXA2	annexin A2 (ANXA2), transcript variant 2, mRNA [NM_001002857]	NM_001002857	302
A_24_P323114	3.56E-03	-1.43	ANXA2P3	annexin A2 pseudogene 3 (ANXA2P3), non-coding RNA [NR_001446]	NR_001446	305
A_24_P8109	5.72E-04	1.41	ANO9	anoctamin 9 (ANO9), mRNA [NM_001012302]	NM_001012302	338440
A_23_P55373	2.62E-02	-1.58	ALOX15	arachidonate 15-lipoxygenase (ALOX15), mRNA [NM_001140]	NM_001140	246
A_33_P3211864	1.75E-03	1.42	ARMCX4	armadillo repeat containing, X-linked 4 (ARMCX4), transcript variant 2, non-coding RNA [NR_028407]	NR_028407	100131755
A_23_P146325	4.81E-02	1.41	ASAP1-IT1	ASAP1 intronic transcript 1 (non-protein coding) (ASAP1-IT1), long non-coding RNA [NR_002765]	NR_002765	29065
A_23_P98686	3.36E-04	1.56	ATHL1	ATH1, acid trehalase-like 1 (yeast) (ATHL1), mRNA [NM_025092]	NM_025092	80162
A_33_P3332414	1.47E-03	1.52	ABCB1	ATP-binding cassette, sub-family B (MDR/TAP), member 1 (ABCB1), mRNA [NM_000927]	NM_000927	5243
A_23_P379034	2.19E-05	1.45	BAIAP2L2	BAI1-associated protein 2-like 2 (BAIAP2L2), mRNA [NM_025045]	NM_025045	80115
A_23_P71946	2.19E-04	1.50	BSPRY	B-box and SPRY domain containing (BSPRY), mRNA [NM_017688]	NM_017688	54836
A_24_P113131	7.04E-03	1.45	BZRAP1	benzodiazapine receptor (peripheral) associated protein 1 (BZRAP1), transcript variant 1, mRNA [NM_004758]	NM_004758	9256
A_23_P92093	2.91E-05	1.62	CELSR3	cadherin, EGF LAG seven-pass G-type receptor 3 (CELSR3), mRNA [NM_001407]	NM_001407	1951
A_33_P3228612	1.32E-02	1.44	CACNA1E	calcium channel, voltage-dependent, R type, alpha 1E subunit (CACNA1E), transcript variant 1, mRNA [NM_001205293]	NM_001205293	777
A_33_P3218960	7.19E-04	1.44	CACNA1H	calcium channel, voltage-dependent, T type, alpha 1H subunit (CACNA1H), transcript variant 1, mRNA [NM_021098]	NM_021098	8912
A_23_P83838	4.94E-02	1.40	CA8	carbonic anhydrase VIII (CA8), mRNA [NM_004056]	NM_004056	767
A_23_P104563	2.73E-03	-1.42	CPT1A	carnitine palmitoyltransferase 1A (liver) (CPT1A), transcript variant 2, mRNA [NM_001031847]	NM_001031847	1374
A_23_P338479	1.34E-03	1.62	CD274	CD274 molecule (CD274), transcript variant 1, mRNA [NM_014143]	NM_014143	29126
A_23_P111583	7.44E-05	-1.56	CD36	CD36 molecule (thrombospondin receptor) (CD36), transcript variant 2, mRNA [NM_001001547]	NM_001001547	948
A_23_P76364	4.01E-04	-1.90	CD9	CD9 molecule (CD9), mRNA [NM_001769]	NM_001769	928
A_32_P170397	9.33E-05	1.51	LOC100289090	cDNA FLJ33063 fis, clone TRACH2000047. [AK057625]	AK057625	100289090
A_33_P3589543	3.24E-04	1.60	LOC692247	cDNA FLJ36520 fis, clone TRACH2002100. [AK093839]	AK093839	692247
A_33_P3362737	2.58E-02	-1.59		cDNA FLJ42705 fis, clone BRAMY3006297. [AK124695]	AK124695	
A_23_P12082	4.12E-03	1.49	CHI3L2	chitinase 3-like 2 (CHI3L2), transcript variant 3, mRNA [NM_001025199]	NM_001025199	1117
A_23_P430792	4.36E-03	1.51	C17orf66	chromosome 17 open reading frame 66 (C17orf66), mRNA [NM_152781]	NM_152781	256957
A_33_P3212799	1.65E-03	1.43	C22orf34	chromosome 22 open reading frame 34 (C22orf34), long non-coding RNA [NR_026997]	NR_026997	348645
A_33_P3422374	4.00E-06	1.81	C6orf163	chromosome 6 open reading frame 163 (C6orf163), mRNA [NM_001010868]	NM_001010868	206412
A_33_P3460043	2.17E-02	-1.40	C8orf56	chromosome 8 open reading frame 56 (C8orf56), long non-coding RNA [NR_027071]	NR_027071	157556
A_32_P101352	1.45E-05	1.49	CXorf65	chromosome X open reading frame 65 (CXorf65), transcript variant 1, mRNA [NM_001025265]	NM_001025265	158830
A_23_P134854	8.06E-03	-1.48	CLDN23	claudin 23 (CLDN23), mRNA [NM_194284]	NM_194284	137075
A_32_P140139	1.14E-02	-1.40	F13A1	coagulation factor XIII, A1 polypeptide (F13A1), mRNA [NM_000129]	NM_000129	2162
A_32_P213661	2.92E-04	1.42	CCDC122	coiled-coil domain containing 122 (CCDC122), mRNA [NM_144974]	NM_144974	160857
A_23_P25674	4.58E-04	-1.48	CKB	creatine kinase, brain (CKB), mRNA [NM_001823]	NM_001823	1152
A_23_P2616	3.16E-02	-1.46	CLEC4C	C-type lectin domain family 4, member C (CLEC4C), transcript variant 1, mRNA [NM_130441]	NM_130441	170482
A_23_P139486	6.94E-05	-1.40	CDK2AP1	cyclin-dependent kinase 2 associated protein 1 (CDK2AP1), transcript variant 1, mRNA [NM_004642]	NM_004642	8099
A_23_P428129	2.97E-03	-1.46	CDKN1C	cyclin-dependent kinase inhibitor 1C (p57, Kip2) (CDKN1C), transcript variant 1, mRNA [NM_000076]	NM_000076	1028
A_23_P2181	7.42E-04	-1.68	CYB5R2	cytochrome b5 reductase 2 (CYB5R2), mRNA [NM_016229]	NM_016229	51700
A_23_P98645	9.00E-03	1.41	DCHS1	dachsous cadherin-related 1 (DCHS1), mRNA [NM_003737]	NM_003737	8642
A_23_P156445	1.33E-03	2.16	DDX43	DEAD (Asp-Glu-Ala-Asp) box polypeptide 43 (DDX43), mRNA [NM_018665]	NM_018665	55510

A_23_P131139	4.36E-02	-1.43	DIRC1	disrupted in renal carcinoma 1 (DIRC1), mRNA [NM_052952]	NM_052952	116093
A_23_P417921	2.79E-02	-1.41	DNAJC5G	DnaJ (Hsp40) homolog, subfamily C, member 5 gamma (DNAJC5G), mRNA [NM_173650]	NM_173650	285126
A_23_P316472	1.24E-04	1.44	DNHD1	dynein heavy chain domain 1 (DNHD1), transcript variant 1, mRNA [NM_144666]	NM_144666	144132
A_33_P3366073	1.79E-04	1.48	EBPL	emopamil binding protein-like (EBPL), transcript variant 3, non-coding RNA [NR_103802]	NR_103802	84650
A_24_P215240	2.83E-02	-1.47	ENKUR	enkurin, TRPC channel interacting protein (ENKUR), transcript variant 1, mRNA [NM_145010]	NM_145010	219670
A_23_P105794	2.82E-03	1.43	EPSTI1	epithelial stromal interaction 1 (breast) (EPSTI1), transcript variant 2, mRNA [NM_033255]	NM_033255	94240
A_33_P3270657	1.27E-03	1.46	FAM111B	family with sequence similarity 111, member B (FAM111B), transcript variant 1, mRNA [NM_198947]	NM_198947	374393
A_32_P108254	7.51E-03	-1.43	FAM20A	family with sequence similarity 20, member A (FAM20A), transcript variant 1, mRNA [NM_017565]	NM_017565	54757
A_23_P431305	2.97E-03	-1.41	FAM69B	family with sequence similarity 69, member B (FAM69B), mRNA [NM_152421]	NM_152421	138311
A_23_P158925	4.71E-04	1.67	GPR125	G protein-coupled receptor 125 (GPR125), mRNA [NM_145290]	NM_145290	166647
A_33_P3550894	8.75E-03	1.79	GATA2	GATA binding protein 2 (GATA2), transcript variant 1, mRNA [NM_001145661]	NM_001145661	2624
A_33_P3334895	4.95E-02	-1.41	GRIN2A	glutamate receptor, ionotropic, N-methyl D-aspartate 2A (GRIN2A), transcript variant 3, mRNA [NM_001134408]	NM_001134408	2903
A_23_P115407	1.02E-03	-1.44	GSTM1	glutathione S-transferase mu 1 (GSTM1), transcript variant 2, mRNA [NM_146421]	NM_146421	2944
A_23_P217917	7.62E-05	-1.48	GSTM4	glutathione S-transferase mu 4 (GSTM4), transcript variant 2, mRNA [NM_147148]	NM_147148	2948
A_23_P7957	2.36E-04	1.41	GNMT	glycine N-methyltransferase (GNMT), mRNA [NM_018960]	NM_018960	27232
A_23_P358195	2.79E-02	1.42	GOLGA6L10	golgin A6 family-like 10 (GOLGA6L10), mRNA [NM_001164465]	NM_001164465	647042
A_33_P3243878	3.17E-04	1.42	GOLGA6L4	golgin A6 family-like 4 (GOLGA6L4), mRNA [NM_001267536]	NM_001267536	643707
A_33_P3220025	1.34E-02	1.72	GOLGA6L6	golgin A6 family-like 6 (GOLGA6L6), mRNA [NM_001145004]	NM_001145004	727832
A_24_P410516	3.57E-04	1.40	GOLGA6L9	golgin A6 family-like 9 (GOLGA6L9), mRNA [NM_198181]	NM_198181	440295
A_23_P156218	5.97E-03	1.54	GZMK	granzyme K (granzyme 3; tryptase II) (GZMK), mRNA [NM_002104]	NM_002104	3003
A_23_P51487	1.89E-02	1.90	GBP3	guanylate binding protein 3 (GBP3), mRNA [NM_018284]	NM_018284	2635
A_23_P74290	3.89E-04	1.51	GBP5	guanylate binding protein 5 (GBP5), transcript variant 1, mRNA [NM_052942]	NM_052942	115362
A_33_P3308481	1.08E-04	1.58	HYMAI	hydatidiform mole associated and imprinted (non-protein coding) (HYMAI), long non-coding RNA [NR_002768]	NR_002768	57061
A_33_P3287428	1.74E-03	1.53	LRRC37A4P	hypothetical protein LOC652203, mRNA (cDNA clone MGC:111480 IMAGE:5581582), complete cds. [BC107850]	BC107850	55073
A_33_P3842551	3.65E-03	1.48	IKZF2	IKAROS family zinc finger 2 (Helios) (IKZF2), transcript variant 2, mRNA [NM_001079526]	NM_001079526	22807
A_33_P3290403	2.04E-04	-1.44	IMPA2	inositol(myo)-1(or 4)-monophosphatase 2 (IMPA2), mRNA [NM_014214]	NM_014214	3613
A_23_P150609	4.73E-02	-1.61	IGF2	insulin-like growth factor 2 (somatomedin A) (IGF2), transcript variant 1, mRNA [NM_000612]	NM_000612	3481
A_33_P3216232	1.04E-02	-1.43	ITGB1BP1	integrin beta 1 binding protein 1 (ITGB1BP1), transcript variant 1, mRNA [NM_004763]	NM_004763	9270
A_23_P45871	3.22E-02	1.44	IFI44L	interferon-induced protein 44-like (IFI44L), mRNA [NM_006820]	NM_006820	10964
A_23_P28334	1.14E-02	1.43	IL18RAP	interleukin 18 receptor accessory protein (IL18RAP), mRNA [NM_003853]	NM_003853	8807
A_33_P3221960	2.31E-02	1.40	IL18RAP	interleukin 18 receptor accessory protein (IL18RAP), mRNA [NM_003853]	NM_003853	8807
A_33_P3328254	5.33E-03	-1.69	IL5RA	interleukin 5 receptor, alpha (IL5RA), transcript variant 3, mRNA [NM_175725]	NM_175725	3568
A_33_P3411315	2.36E-02	-1.42	KRTAP3-3	keratin associated protein 3-3 (KRTAP3-3), mRNA [NM_033185]	NM_033185	85293
A_33_P3265222	2.43E-02	1.60	KIAA1324	KIAA1324 (KIAA1324), transcript variant 1, mRNA [NM_020775]	NM_020775	57535
A_33_P3883985	3.66E-04	1.74	LMF1	lipase maturation factor 1 (LMF1), transcript variant 4, non-coding RNA [NR_036442]	NR_036442	64788
A_32_P324933	6.55E-04	1.55	LINC00282	long intergenic non-protein coding RNA 282 (LINC00282), transcript variant 1, long non-coding RNA [NR_027047]	NR_027047	283521
A_33_P3390177	4.26E-02	-1.43	LINC00884	long intergenic non-protein coding RNA 884 (LINC00884), long non-coding RNA [NR_033929]	NR_033929	401106
A_33_P3226395	1.26E-02	1.45	LINC00937	long intergenic non-protein coding RNA 937 (LINC00937), long non-coding RNA [NR_024420]	NR_024420	389634
A_32_P221305	1.53E-02	1.42	LINC00937	long intergenic non-protein coding RNA 937 (LINC00937), long non-coding RNA [NR_024420]	NR_024420	389634
A_23_P94186	4.20E-03	-1.73	LYPD2	LY6/PLAUR domain containing 2 (LYPD2), mRNA [NM_205545]	NM_205545	137797
A_23_P101992	4.37E-03	-1.63	MARCO	macrophage receptor with collagenous structure (MARCO), mRNA [NM_006770]	NM_006770	8685
A_33_P3424222	2.95E-03	-1.61	HLA-DQB1	major histocompatibility complex, class II, DQ beta 1 (HLA-DQB1), transcript variant 1, mRNA [NM_002123]	NM_002123	3119
A_23_P136683	6.20E-03	-2.16	HLA-DQB1	major histocompatibility complex, class II, DQ beta 1 (HLA-DQB1), transcript variant 2, mRNA [NM_001243961]	NM_001243961	3119
A_33_P3424217	8.09E-04	-1.54	HLA-DQB1	major histocompatibility complex, class II, DQ beta 1 (HLA-DQB1), transcript variant 3, mRNA [NM_001243962]	NM_001243962	3119
A_23_P19510	7.06E-03	-1.44	HLA-DQB2	major histocompatibility complex, class II, DQ beta 2 (HLA-DQB2), mRNA [NM_001198858]	NM_001198858	3120
A_23_P116614	3.12E-03	1.45	ME3	malic enzyme 3, NADP(+)-dependent, mitochondrial (ME3), transcript variant 2, mRNA [NM_001014811]	NM_001014811	10873
A_33_P3236881	7.97E-03	1.46	MINOS1-NBL1	MINOS1-NBL1 readthrough (MINOS1-NBL1), transcript variant 1, mRNA [NM_001204088]	NM_001204088	100532736
A_33_P3350202	2.52E-02	-1.46	MOCS3	molybdenum cofactor synthesis 3 (MOCS3), mRNA [NM_014484]	NM_014484	27304
A_33_P3281795	4.37E-03	-1.47	MGLL	monoglyceride lipase (MGLL), transcript variant 1, mRNA [NM_007283]	NM_007283	11343

A_32_P216872	1.06E-03	1.41	PDXDC2P	mRNA; cDNA DKFZp686B0962 (from clone DKFZp686B0962). [BX647358]	BX647358	283970
A_33_P3514859	1.10E-03	1.41	LOC100506342	mRNA; cDNA DKFZp686L14188 (from clone DKFZp686L14188). [BX538329]	BX538329	100506342
A_23_P153616	3.16E-02	-1.52	MADCAM1	mucosal vascular addressin cell adhesion molecule 1 (MADCAM1), transcript variant 1, mRNA [NM_130760]	NM_130760	8174
A_24_P926960	3.84E-02	1.42	MEGF6	multiple EGF-like-domains 6 (MEGF6), mRNA [NM_001409]	NM_001409	1953
A_24_P88850	9.76E-04	-1.43	MRAS	muscle RAS oncogene homolog (MRAS), transcript variant 1, mRNA [NM_012219]	NM_012219	22808
A_24_P134195	4.03E-03	-1.41	MYADM	myeloid-associated differentiation marker (MYADM), transcript variant 1, mRNA [NM_001020818]	NM_001020818	91663
A_23_P148737	1.33E-02	1.49	MYBPH	myosin binding protein H (MYBPH), mRNA [NM_004997]	NM_004997	4608
A_32_P475513	1.43E-05	1.46	MYO15B	myosin XVB pseudogene (MYO15B), non-coding RNA [NR_003587]	NR_003587	80022
A_23_P210425	2.00E-02	-1.66	MYL9	myosin, light chain 9, regulatory (MYL9), transcript variant 2, mRNA [NM_181526]	NM_181526	10398
A_33_P3325275	5.47E-03	-1.44	NRSN2	neurensin 2 (NRSN2), mRNA [NM_024958]	NM_024958	80023
A_24_P273157	1.02E-04	1.44	OBSCN	obscurin, cytoskeletal calmodulin and titin-interacting RhoGEF (OBSCN), transcript variant 1, mRNA [NM_052843]	NM_052843	84033
A_23_P123172	2.41E-02	1.40	OR2A9P	olfactory receptor, family 2, subfamily A, member 9 pseudogene (OR2A9P), non-coding RNA [NR_002157]	NR_002157	441295
A_33_P3319834	4.21E-02	-1.82	OR2T8	olfactory receptor, family 2, subfamily T, member 8 (OR2T8), mRNA [NM_001005522]	NM_001005522	343172
A_23_P154849	4.95E-03	-1.40	OLIG1	oligodendrocyte transcription factor 1 (OLIG1), mRNA [NM_138983]	NM_138983	116448
A_33_P3215640	1.51E-02	1.44	PI16	peptidase inhibitor 16 (PI16), transcript variant 1, mRNA [NM_153370]	NM_153370	221476
A_33_P3358213	2.96E-04	-2.13	PADI6	peptidyl arginine deiminase, type VI (PADI6), mRNA [NM_207421]	NM_207421	353238
A_23_P42144	4.12E-05	-2.26	PEX6	peroxisomal biogenesis factor 6 (PEX6), mRNA [NM_000287]	NM_000287	5190
A_33_P3265016	3.69E-04	-1.82	PEX6	peroxisomal biogenesis factor 6 (PEX6), mRNA [NM_000287]	NM_000287	5190
A_23_P157736	2.26E-04	-1.45	PPAPDC3	phosphatidic acid phosphatase type 2 domain containing 3 (PPAPDC3), mRNA [NM_032728]	NM_032728	84814
A_32_P94667	1.61E-02	1.59	PDE4DIP	phosphodiesterase 4D interacting protein (PDE4DIP), transcript variant 1, mRNA [NM_014644]	NM_014644	9659
A_33_P3326992	9.37E-03	1.55	PDE4DIP	phosphodiesterase 4D interacting protein (PDE4DIP), transcript variant 1, mRNA [NM_014644]	NM_014644	9659
A_33_P3323803	5.32E-03	1.42	PDE4DIP	phosphodiesterase 4D interacting protein (PDE4DIP), transcript variant 1, mRNA [NM_014644]	NM_014644	9659
A_33_P3389649	2.55E-02	1.42	PDE4D	phosphodiesterase 4D, cAMP-specific (PDE4D), transcript variant 4, mRNA [NM_001197218]	NM_001197218	5144
A_32_P78816	4.78E-02	1.90	PSPH	phosphoserine phosphatase (PSPH), mRNA [NM_004577]	NM_004577	5723
A_33_P3317628	4.43E-03	1.40	PKP3	plakophilin 3 (PKP3), mRNA [NM_007183]	NM_007183	11187
A_33_P3413048	2.60E-04	1.43	PLXNA3	plexin A3 (PLXNA3), mRNA [NM_017514]	NM_017514	55558
A_33_P3253672	4.43E-02	-1.46	KCNH3	potassium voltage-gated channel, subfamily H (eag-related), member 3 (KCNH3), mRNA [NM_012284]	NM_012284	23416
A_33_P3312779	2.44E-02	-1.68	PRSS33	protease, serine, 33 (PRSS33), mRNA [NM_152891]	NM_152891	260429
A_23_P203475	5.88E-03	-1.46	PRKCDBP	protein kinase C, delta binding protein (PRKCDBP), mRNA [NM_145040]	NM_145040	112464
A_33_P3335682	1.99E-02	-1.45	PPP1R14A	protein phosphatase 1, regulatory (inhibitor) subunit 14A (PPP1R14A), transcript variant 1, mRNA [NM_033256]	NM_033256	94274
A_23_P145724	3.22E-03	-1.74	PPP1R17	protein phosphatase 1, regulatory subunit 17 (PPP1R17), transcript variant 1, mRNA [NM_006658]	NM_006658	10842
A_24_P243749	8.37E-03	-1.67	PDK4	pyruvate dehydrogenase kinase, isozyme 4 (PDK4), mRNA [NM_002612]	NM_002612	5166
A_24_P218814	1.03E-05	1.50	RDH5	retinol dehydrogenase 5 (11-cis/9-cis) (RDH5), transcript variant 2, mRNA [NM_002905]	NM_002905	5959
A_23_P75310	2.41E-04	-1.51	ARHGAP22	Rho GTPase activating protein 22 (ARHGAP22), transcript variant 3, mRNA [NM_021226]	NM_021226	58504
A_33_P3299110	3.51E-02	-1.48	ARHGAP42	Rho GTPase activating protein 42 (ARHGAP42), mRNA [NM_152432]	NM_152432	143872
A_33_P3387616	4.32E-03	1.43	RHPN1	rhophilin, Rho GTPase binding protein 1 (RHPN1), mRNA [NM_052924]	NM_052924	114822
A_33_P3413989	6.12E-03	1.46	SERPING1	serpin peptidase inhibitor, clade G (C1 inhibitor), member 1 (SERPING1), transcript variant 1, mRNA [NM_000062]	NM_000062	710
A_33_P3382498	6.37E-03	7.57	SIGLEC14	sialic acid binding Ig-like lectin 14 (SIGLEC14), mRNA [NM_001098612]	NM_001098612	100049587
A_24_P274270	1.16E-03	1.42	STAT1	signal transducer and activator of transcription 1, 91kDa (STAT1), transcript variant beta, mRNA [NM_139266]	NM_139266	6772
A_33_P3248072	2.75E-02	2.10	SIRPB1	signal-regulatory protein beta 1 (SIRPB1), transcript variant 3, mRNA [NM_001135844]	NM_001135844	10326
A_24_P785293	1.33E-03	1.41	SNHG1	small nucleolar RNA host gene 1 (non-protein coding) (SNHG1), long non-coding RNA [NR_003098]	NR_003098	23642
A_23_P37914	1.26E-02	1.74	SLC5A11	solute carrier family 5 (sodium/inositol cotransporter), member 11 (SLC5A11), transcript variant 1, mRNA [NM_052944	NM_052944	115584
A_23_P320113	2.15E-04	-1.50	SRXN1	sulfiredoxin 1 (SRXN1), mRNA [NM_080725]	NM_080725	140809
A_23_P163697	1.66E-03	-1.43	SYT17	synaptotagmin XVII (SYT17), mRNA [NM_016524]	NM_016524	51760
A_32_P231179	7.87E-03	-1.47	TEKT4	tektin 4 (TEKT4), transcript variant 1, mRNA [NM_144705]	NM_144705	150483
A_24_P306443	3.04E-02	-1.45	LOC100233156	tektin 4 pseudogene (LOC100233156), transcript variant 1, non-coding RNA [NR_037871]	NR_037871	100233156
A_24_P287189	6.60E-03	-1.77	TOLLIP	toll interacting protein (TOLLIP), mRNA [NM_019009]	NM_019009	54472
A_33_P3387696	1.79E-02	-1.59	TMBIM4	transmembrane BAX inhibitor motif containing 4 (TMBIM4), transcript variant 1, mRNA [NM_001282606]	NM_001282606	51643
A_33_P3411632	1.36E-03	1.47	TMEM121	transmembrane protein 121 (TMEM121), mRNA [NM_025268]	NM_025268	80757

A_33_P3338417	1.79E-02	1.47	WARS2	tryptophanyl tRNA synthetase 2, mitochondrial (WARS2), transcript variant 2, mRNA [NM_201263]	NM_201263	10352
A_23_P26386	5.26E-03	-1.49	TPPP3	tubulin polymerization-promoting protein family member 3 (TPPP3), mRNA [NM_016140]	NM_016140	51673
A_23_P19291	3.79E-03	3.03	TUBB2A	tubulin, beta 2A class IIa (TUBB2A), mRNA [NM_001069]	NM_001069	7280
A_23_P254271	2.04E-02	-1.40	TUBB6	tubulin, beta 6 class V (TUBB6), mRNA [NM_032525]	NM_032525	84617
A_24_P813147	4.62E-02	1.77	TUBB8	tubulin, beta 8 class VIII (TUBB8), transcript variant 1, mRNA [NM_177987]	NM_177987	347688
A_23_P30163	6.95E-03	1.46	FLJ13197	uncharacterized FLJ13197 (FLJ13197), long non-coding RNA [NR_026804]	NR_026804	79667
A_33_P3229863	1.41E-02	-1.43	LOC100128714	uncharacterized LOC100128714 (LOC100128714), long non-coding RNA [NR_040082]	NR_040082	100128714
A_33_P3409580	4.99E-02	-1.69	LOC100129722	uncharacterized LOC100129722 (LOC100129722), long non-coding RNA [NR_038389]	NR_038389	100129722
A_33_P3327697	2.38E-03	1.43	LOC145474	uncharacterized LOC145474 (LOC145474), long non-coding RNA [NR_027046]	NR_027046	145474
A_32_P153071	1.36E-03	1.55	VIPR2	vasoactive intestinal peptide receptor 2 (VIPR2), mRNA [NM_003382]	NM_003382	7434
A_23_P105562	3.82E-03	-1.71	VWF	von Willebrand factor (VWF), mRNA [NM_000552]	NM_000552	7450
A_23_P42288	1.63E-02	-1.44	VWA7	von Willebrand factor A domain containing 7 (VWA7), mRNA [NM_025258]	NM_025258	80737
A_32_P80245	8.82E-03	2.74	ZFP57	ZFP57 zinc finger protein (ZFP57), mRNA [NM_001109809]	NM_001109809	346171
A_23_P131024	1.18E-02	1.41	ZBTB32	zinc finger and BTB domain containing 32 (ZBTB32), mRNA [NM_014383]	NM_014383	27033
A_33_P3424339	1.51E-02	-1.41		1203217A dehydrogenase,glyceraldehydephosphate. [THC2554230]		
A_24_P461497	9.40E-03	-1.42		Actin beta pseudogene (LOC100418883) on Chromosome 19 [NG_0242		
A_33_P3407049	1.37E-03	1.42		BAC clone CH17-90K13 from chromosome 7	AC254813	
A_33_P3362886	3.55E-02	1.44		cDNA FLJ31857 fis	AK056419	
A_33_P3267118	7.91E-03	1.76	EIF4G3	eukaryotic translation initiation factor 4 gamma, 3 [Source:HGNC Symbol;Acc:3298] [ENST00000374933]		8672
A_33_P3414880	3.33E-07	1.51		FMNL_HUMAN (O95466) Formin-like 1 protein (Formin-like protein) (Leukocyte formin) (CLL-associated antigen KW-13), partial (3%) [THC2502506]		
A_33_P3351180	3.34E-02	1.50		immunoglobulin heavy constant alpha 1 [Source:HGNC Symbol;Acc:5478] [ENST00000390547]	S55735	
A_33_P3424591	2.01E-02	1.55		immunoglobulin heavy constant gamma 1 (G1m marker) [Source:HGNC Symbol;Acc:5525] [ENST00000390549]	BC073773	
A_33_P3263319	2.76E-02	1.60		immunoglobulin heavy constant gamma 3 (G3m marker) [Source:HGNC Symbol;Acc:5527] [ENST00000390551]	AY172958	
A_33_P3238820	3.62E-05	-1.89		LINC rna TCONS_l2_00011463	TCONS_l2_00011463	
A_33_P3387352	1.85E-03	-2.43		major histocompatibility complex, class I, V (pseudogene) [Source:HGNC Symbol;Acc:23482] [ENST00000411514]	BC068238	
A_33_P3299796	9.59E-04	1.47	MAN1B1	mannosidase, alpha, class 1B, member 1 [Source:HGNC Symbol;Acc:6823] [ENST00000536349]	XM_005276366	11253
A_33_P3326733	4.16E-04	1.41	MTPAP	mitochondrial poly(A) polymerase [Source:HGNC Symbol;Acc:25532] [ENST00000471055]	XR_171057	55149
A_33_P3263538	1.75E-03	1.66		nuclear paraspeckle assembly transcript 1 (non-protein coding) [Source:HGNC Symbol;Acc:30815] [ENST00000501122]	AF001893	
A_33_P3300495	3.56E-03	1.80		plasminogen-like B1 [Source:HGNC Symbol;Acc:9072] [ENST00000409310]	BC022294	
A_33_P3340324	2.19E-03	1.51	LOC345051	PREDICTED: hCG38984 (LOC345051), transcript variant X1, misc_RNA [XR_109844]	XR_109844	345051
A_33_P3388771	2.33E-02	4.76	LOC101060810	PREDICTED: zinc finger protein 98-like (LOC101060810), misc_RNA [XR_172438]	XR_172438	101060810
A_33_P3398143	1.01E-03	-3.85		Q59H50_HUMAN (Q59H50) Integrin beta chain, beta 2 variant (Fragment), partial (11%) [THC2546843]		
A_24_P24972	3.94E-02	-1.41		Q7Z4W8_HUMAN (Q7Z4W8) Heparin-binding protein HBp15, partial (92%) [THC2549395]		
A_33_P3292874	8.34E-04	1.52	ARHGEF1	Rho guanine nucleotide exchange factor (GEF) 1 [Source:HGNC Symbol;Acc:681] [ENST00000595897]	AK090448	9138
A_33_P3351351	4.27E-02	-1.41	ARHGEF10	Rho guanine nucleotide exchange factor (GEF) 10 [Source:HGNC Symbol;Acc:14103] [ENST00000382795]	BC040474	9639
A_33_P3259705	8.88E-06	1.55	SIGIRR	single immunoglobulin and toll-interleukin 1 receptor (TIR) domain [Source:HGNC Symbol;Acc:30575] [ENST0000038252	XM_005253044	59307
A_33_P3264569	3.07E-02	1.66		T cell receptor beta variable 10-2 [Source:HGNC Symbol;Acc:12178] [ENST00000426318]	AB306184	
A_33_P3416347	1.43E-02	1.54		T cell receptor beta variable 23-1 (non-functional) [Source:HGNC Symbol;Acc:12201] [ENST00000390396]	M27390	
A_33_P3374162	1.58E-04	1.53		T cell receptor beta variable 27 [Source:HGNC Symbol;Acc:12208] [ENST00000390399]	AB305921	
A_33_P3233404	1.55E-03	2.34		T cell receptor beta variable 28 [Source:HGNC Symbol;Acc:12209] [ENST00000390400]	AB305916	
A_33_P3806676	1.22E-03	2.02		T cell receptor beta variable 28 [Source:HGNC Symbol;Acc:12209] [ENST00000390400]	AY751906	
A_33_P3314850	3.33E-04	1.51		T cell receptor beta variable 6-4 [Source:HGNC Symbol;Acc:12229] [ENST00000390360]	A25966	
A_33_P3244312	4.48E-02	1.72		T cell receptor gamma variable 2 [Source:HGNC Symbol;Acc:12287] [ENST00000426402]	M27337	
A_33_P3398196	4.01E-02	-1.54	USP32	ubiquitin specific peptidase 32 [Source:HGNC Symbol;Acc:19143] [ENST00000585720]		84669
A_33_P3235831	1.22E-02	-1.40		Uncharacterized chromosome 10 clone RP11-351o1 [AC022022.10]		
A_33_P3280502	5.34E-03	1.45		Uncharacterized LOC100505915 [AK125313.1]		
A_33_P3303355	4.63E-02	-1.51	ZCCHC18	zinc finger, CCHC domain containing 18 [Source:HGNC Symbol;Acc:32459] [ENST00000605835]		644353

Supplementary Table 3. LS Gene List. Probes can be identified by Agilent probe ID in cases where GenBank accession is not available.
