# Supplementary figures and images for: Transcriptomic signatures in whole blood of patients who acquire a chronic inflammatory response syndrome (CIRS) following an exposure to the marine toxin ciguatoxin
Source: BMC Med Genomics. 2015 Apr 2;8:15. doi: 10.1186/s12920-015-0089-x (PMC4392619; doi:10.1186/s12920-015-0089-x)

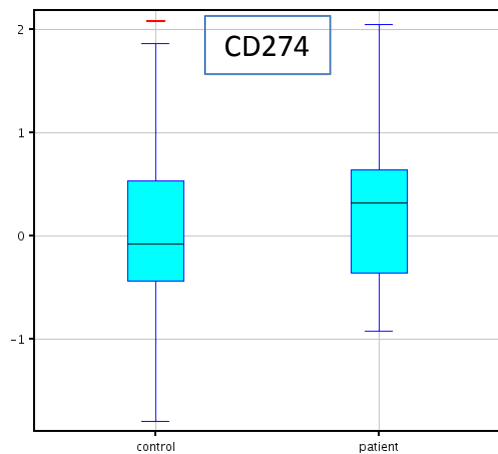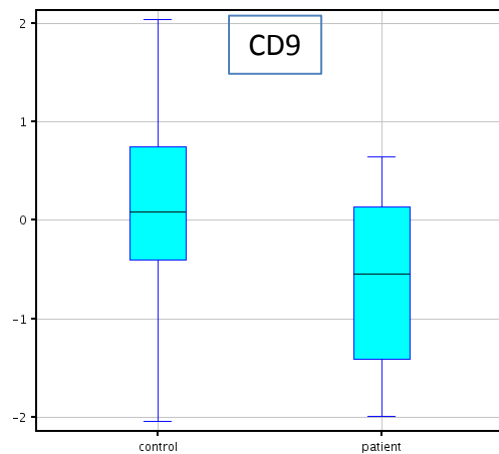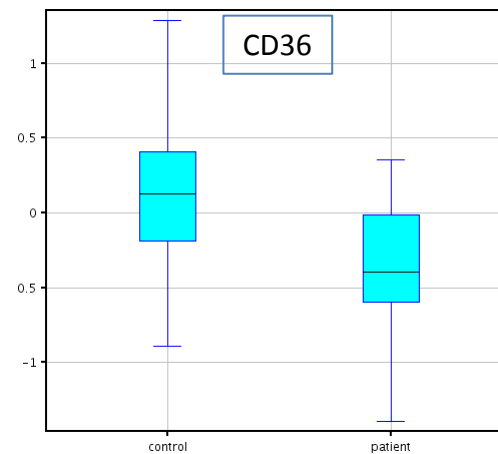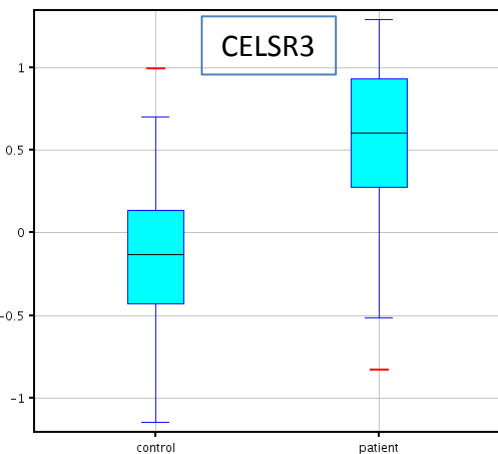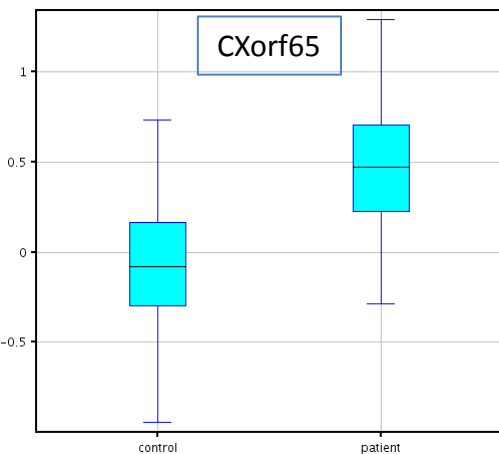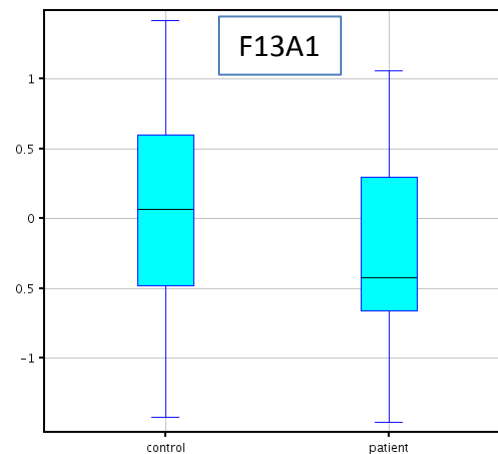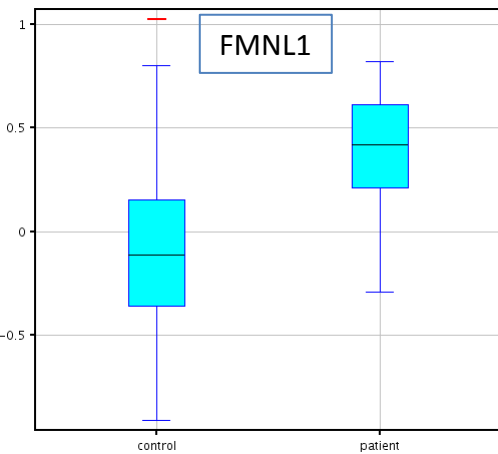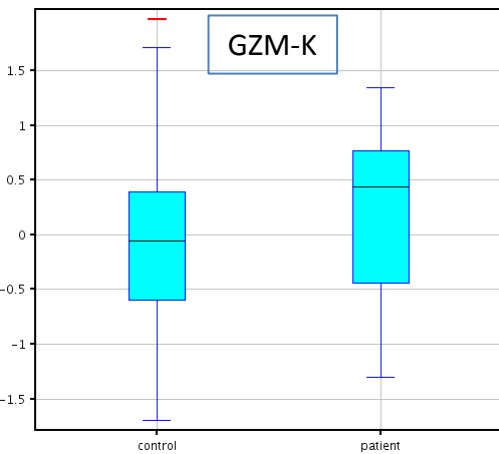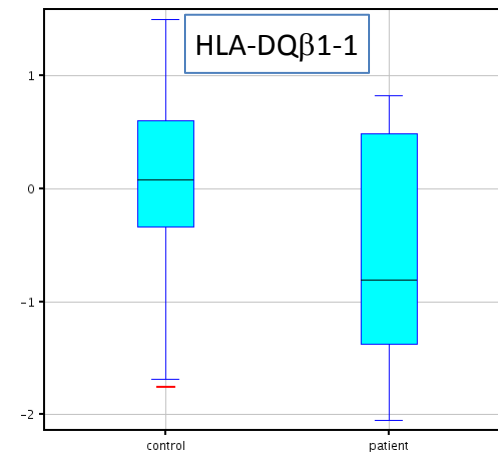

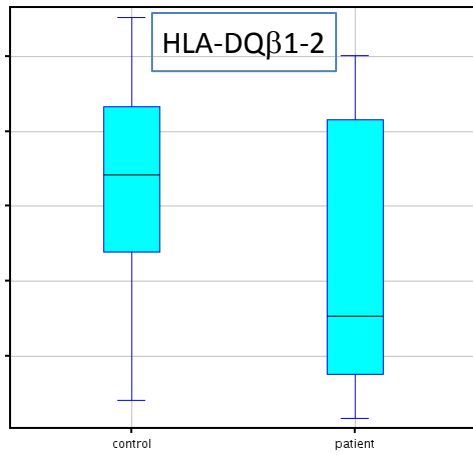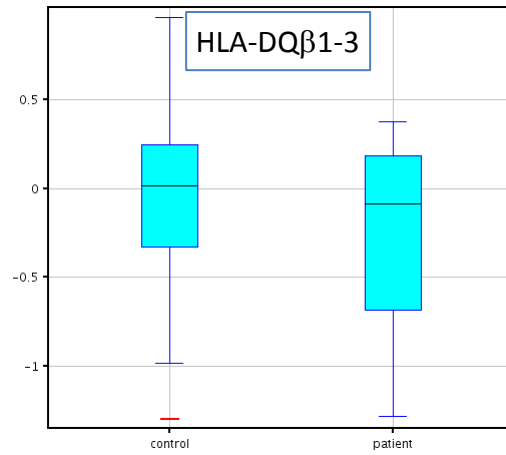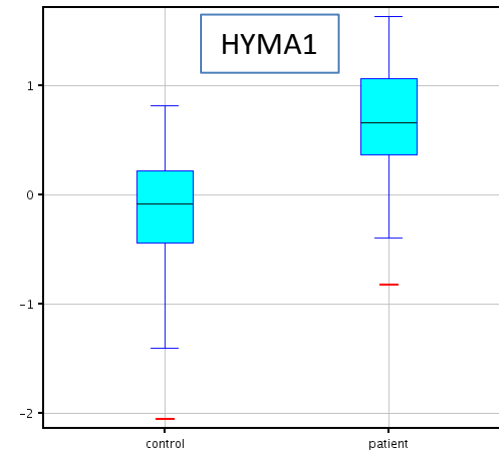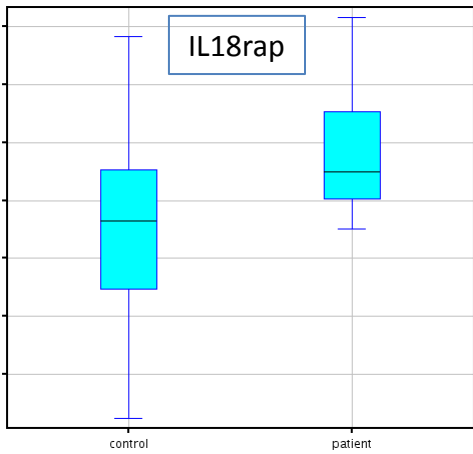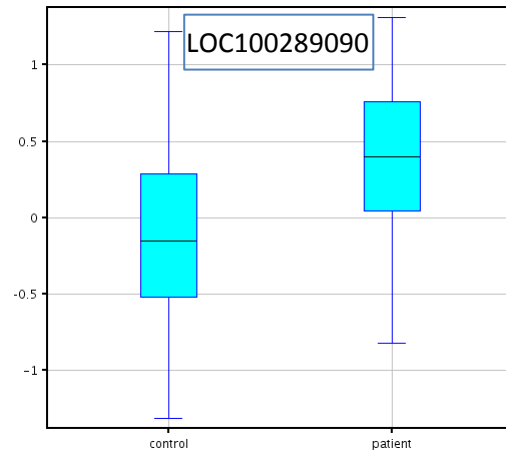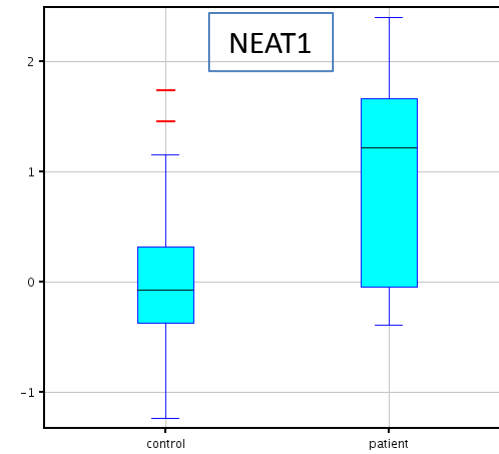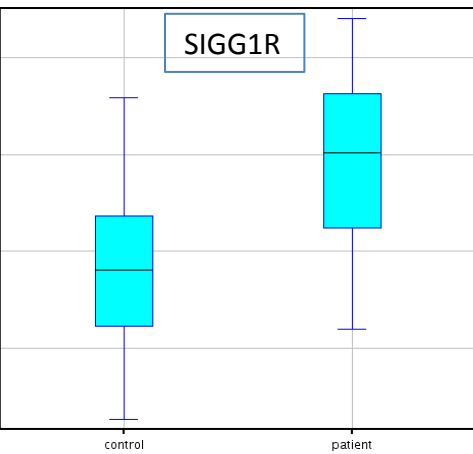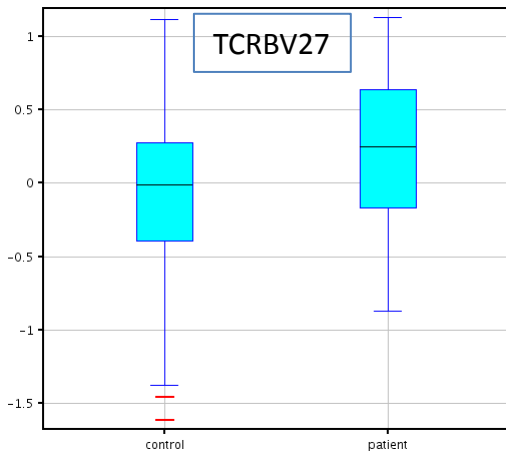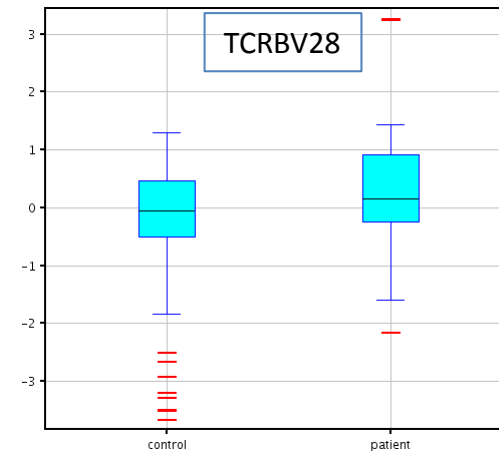

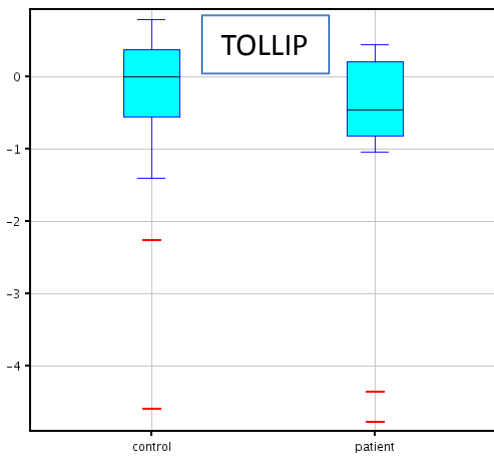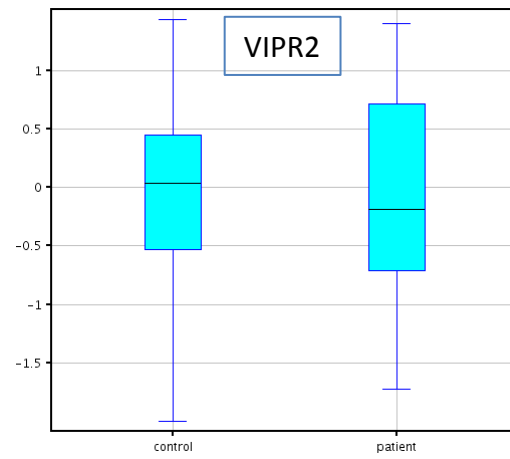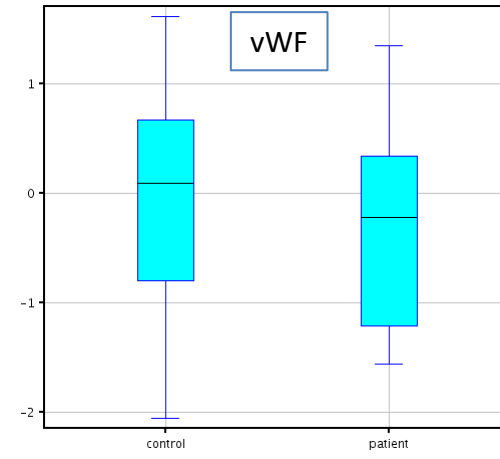

Supplement: Additional file 4: Figure S1. — Gene Expression Analysis with Extended Control Database. Box whisker plots show specific gene expression comparisons between these 11 CIRS ciguatera patients with 79 gene expression profiles from 30 male and 18 female control subjects. The shaded box indicates the interquartile range of values (25% - 75%) while the interior solid line indicates the median. [file 12920_2015_89_MOESM4_ESM.pdf]

# HS

- Control
- Patient

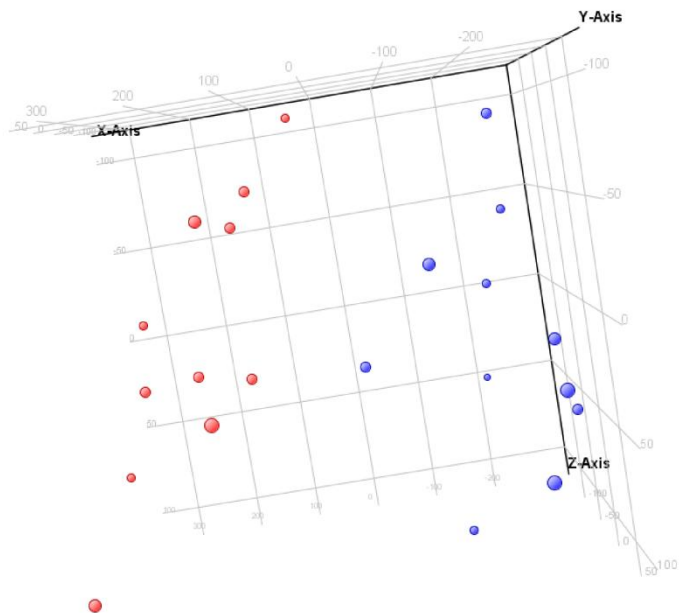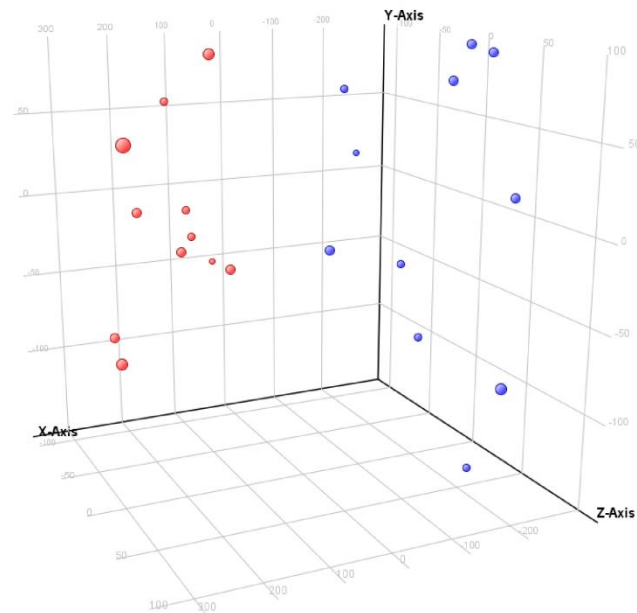

# LS

- Control
- Patient

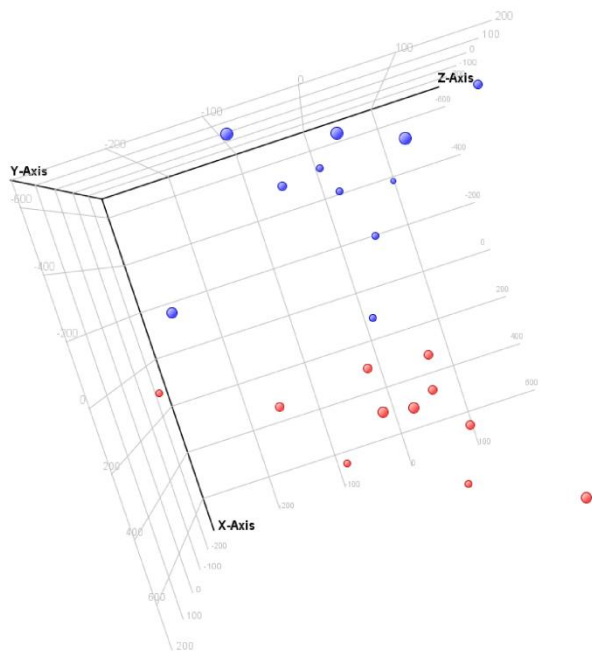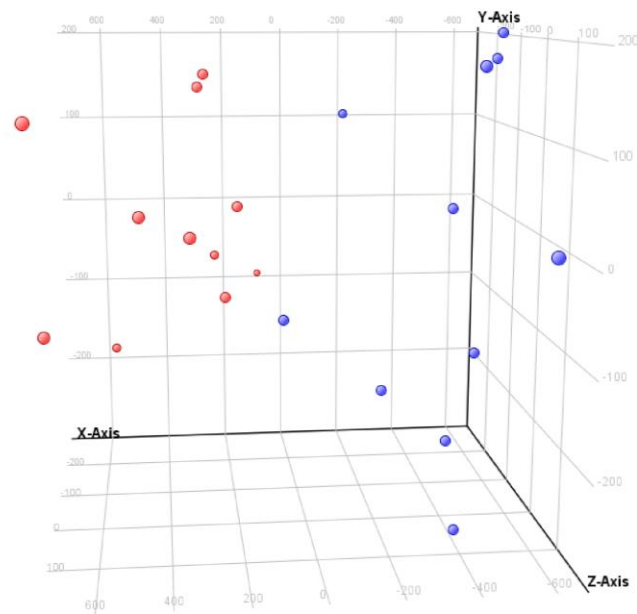

Supplement: Additional file 6: Figure S2. — PCA of averaged replicates. Replicate control and patient profiles were averaged then subjected to principal component analysis. Plots were rotated to angles that visually best exhibit their separation. X, Y and Z axes indicate first three principle components. HS = high stringency, LS = low stringency gene sets. [file 12920_2015_89_MOESM6_ESM.pdf]
