# Supplementary material for: Transcriptomic signatures in whole blood of patients who acquire a chronic inflammatory response syndrome (CIRS) following an exposure to the marine toxin ciguatoxin
Source: BMC Med Genomics. 2015 Apr 2;8:15. doi: 10.1186/s12920-015-0089-x (PMC4392619; doi:10.1186/s12920-015-0089-x)
Supplement: Additional file 5: Table S4. — Primers used in qPCR reactions. PB: PrimerBank: http://pga.mgh.harvard.edu/primerbank/. * denotes transcript used as a reference. [file 12920_2015_89_MOESM5_ESM.pdf]

primer name	sequence (5'-3')	size (bp)	reference	efficiency	R^2
HYMAI-F	GTGGAGCAAAGCTGTTGTTTAG	22	this study	0.989	0.9996
HYMAI-R	GCACAGGTCAGGCGATAAA	19	this study		
VWF-F	AGCCATGTACTCCATTGACATC	22	this study	1.045	0.9987
VWF-R	GGCATTGAGAACCTCATGGTA	21	this study		
CD9-F	TTCCTCTTGGTGATATTCGCCA	22	PB	0.988	0.9935
CD9-R	AGTTCAACGCATAGTGGATGG	21	PB		
CXorf65-F	TGCTTACAGAGCTTTTGTGCC	21	PB	0.9997	0.9967
CXorf65-R	GCGAGTGGACTTCTTTTTGTCT	22	PB		
F13A1-F	GAGCCTTCAGCACTAACCTATG	22	this study	0.987	0.9991
F13A1-R	CTTGGCAAATGCTGTGAGATTAC	23	this study		
IL18RAP-F	TGGGATTGAGACTGTGGTTTAG	22	this study	0.986	0.997
IL18RAP-R	CGGACCACTGGGAAATCTTAG	21	this study		
TCRBV28-F	GTAGGCCTCGTAGATGTGAAAG	22	this study	0.979	0.9991
TCRBV28-R	GGTCCATATCCTGGACACATTC	22	this study		
*CCDC12-F	GAAGGCCCTACGGGAGAAAAC	21	PB	0.976	0.9994
*CCDC12-R	CCGCAGCCTAAGTTCCCTG	19	PB		

Supplementary Table 4. Primers used in qPCR reactions. PB: PrimerBank: http://pga.mgh.harvard.edu/primerbank/. * denotes transcript used as a reference.
