# Supplementary material for: Transcriptomic signatures in whole blood of patients who acquire a chronic inflammatory response syndrome (CIRS) following an exposure to the marine toxin ciguatoxin
Source: BMC Med Genomics. 2015 Apr 2;8:15. doi: 10.1186/s12920-015-0089-x (PMC4392619; doi:10.1186/s12920-015-0089-x)
Supplement: Additional file 7: Table S5. — SVM Classification of averaged replicates. Results of predictions and confidence for the average of duplicate arrays for cases of CIRS-ciguatera and controls using a Support Vector Machines classification algorithm, while withholding four data profiles* from training. [file 12920_2015_89_MOESM7_ESM.pdf]

Identifier	Gender	Trained	Predicted	Confidence
Control 1	Female	control	[control]	0.532
Control 2	Male	control	[control]	0.532
Control 3	Male	control	[control]	0.636
Control 4	Male	control	[control]	0.664
Control 5	Male	control	[control]	1.000
Control 6*	Male		[control]	0.247
Control 7	Female	control	[control]	0.532
Control 8	Male	control	[control]	0.532
Control 9	Male	control	[control]	0.741
Control 10*	Male		[control]	0.496
Control 11	Male	control	[control]	0.532
Patient 1	Male	patient	[patient]	0.577
Patient 2	Male	patient	[patient]	0.648
Patient 3	Male	patient	[patient]	0.874
Patient 4	Female	patient	[patient]	0.577
Patient 5*	Male		[patient]	0.330
Patient 6	Male	patient	[patient]	0.577
Patient 7	Male	patient	[patient]	0.577
Patient 8	Male	patient	[patient]	0.577
Patient 9	Female	patient	[patient]	0.577
Patient 10*	Male		[patient]	0.250
Patient 11	Male	patient	[patient]	1.000

Supplementary Table 5. SVM Classification of Averaged Technical Replicates. Results of predictions and confidence for cases of CIRS-ciguatera and controls using a Support Vector Machines classification algorithm on the averaged technical replicates, while witholding four data profiles* from training.
